# Identification of essential genes of the periodontal pathogen *Porphyromonas gingivalis*

**DOI:** 10.1186/1471-2164-13-578

**Published:** 2012-10-31

**Authors:** Brian A Klein, Elizabeth L Tenorio, David W Lazinski, Andrew Camilli, Margaret J Duncan, Linden T Hu

**Affiliations:** 1 Department of Molecular Biology and Microbiology, Tufts University Sackler School of Biomedical Sciences, Boston, MA, 02111, USA; 2 Division of Geographic Medicine and Infectious Disease, Tufts Medical Center, Boston, MA, 02111, USA; 3 Howard Hughes Medical Institute and Department of Molecular Biology and Microbiology, Tufts University School of Medicine, Boston, MA, 02111, USA; 4 Department of Molecular Genetics, The Forsyth Institute, Cambridge, MA, 02142, USA

**Keywords:** *Porphyromonas gingivalis*, Transposon mutagenesis, Essential genes, Tn-seq, Periodontal disease

## Abstract

**Background:**

*Porphyromonas gingivalis* is a Gram-negative anaerobic bacterium associated with periodontal disease onset and progression. Genetic tools for the manipulation of bacterial genomes allow for in-depth mechanistic studies of metabolism, physiology, interspecies and host-pathogen interactions. Analysis of the essential genes, protein-coding sequences necessary for survival of *P. gingivalis* by transposon mutagenesis has not previously been attempted due to the limitations of available transposon systems for the organism. We adapted a Mariner transposon system for mutagenesis of *P. gingivalis* and created an insertion mutant library. By analyzing the location of insertions using massively-parallel sequencing technology we used this mutant library to define genes essential for *P. gingivalis* survival under *in vitro* conditions.

**Results:**

In mutagenesis experiments we identified 463 genes in *P. gingivalis* strain ATCC 33277 that are putatively essential for viability *in vitro*. Comparing the 463 *P. gingivalis* essential genes with previous essential gene studies, 364 of the 463 are homologues to essential genes in other species; 339 are shared with more than one other species. Twenty-five genes are known to be essential in *P. gingivalis* and *B. thetaiotaomicron* only. Significant enrichment of essential genes within Cluster of Orthologous Groups ‘D’ (cell division), ‘I’ (lipid transport and metabolism) and ‘J’ (translation/ribosome) were identified. Previously, the *P. gingivalis* core genome was shown to encode 1,476 proteins out of a possible 1,909; 434 of 463 essential genes are contained within the core genome. Thus, for the species *P. gingivalis* twenty-two, seventy-seven and twenty-three percent of the genome respectively are devoted to essential, core and accessory functions.

**Conclusions:**

A Mariner transposon system can be adapted to create mutant libraries in *P. gingivalis* amenable to analysis by next-generation sequencing technologies. *In silico* analysis of genes essential for *in vitro* growth demonstrates that although the majority are homologous across bacterial species as a whole, species and strain-specific subsets are apparent. Understanding the putative essential genes of *P. gingivalis* will provide insights into metabolic pathways and niche adaptations as well as clinical therapeutic strategies.

## Background

*Porphyromonas gingivalis* is an oral Gram-negative, anaerobic, asaccharolytic and black-pigmented bacterium that is highly correlated with the development and progression of periodontal diseases and systemic comorbidities [1-5]. The organism has been characterized as a ‘keystone’ pathogen whose interactions with other bacteria and the host are critical for the development of periodontitis [5]. *P. gingivalis* utilizes multiple virulence factors, many of which have been identified and studied *in vitro* and *in vivo* such as proteinases (e.g. gingipains), fimbriae, non-canonical lipopolysaccharide (LPS), capsular polysaccharide (CPS), and cytotoxic and hemolytic molecules [6-8]. The identification of genes and proteins involved in pathogenesis has most commonly relied on analyzing genetic variations between strains or by directly isolating then genetically and biochemically characterizing specific mutants [9-12]. In contrast, the identification of essential genes in this organism has lagged. Essential genes can be used as targets for antimicrobial drug development, and through bioinformatic and experimental study of essential gene, may reveal unique aspects of the physiology and metabolism of *P. gingivalis.* High-throughput strategies to screen for genetic determinants of virulence and identify essential genes have been limited due to a paucity of tools for genetic manipulation [13].

Transposon mutagenesis has been used to identify genes involved in pathogenesis and other bacterial functions [14-18]. The utility of transposon mutagenesis depends on the ability of the transposable element to insert randomly into different sites in the host genome in a one insertion per strain manner. Two previous transposon mutagenesis systems for *P. gingivalis* were based on Tn*4435* and Tn*4400*[19-23]. While the use of the mutant libraries generated with these systems led to important insights into *P. gingivalis* pathogenesis, both elements inserted preferentially into ‘hot-spots’ in the genome thus limiting the distribution of interrupted genes and were also limited to which strains could be mutagenized. The lack of insertion saturation with these transposons into *P. gingivalis* genes resulted in libraries that were not suitable for the genome-wide identification of essential genes.

Mariner-family transposons have been used to generate highly-saturated mutant libraries in numerous phylogenetically distinct bacterial species [17]. Mariner transposons preferentially insert into ‘TA’ nucleotide sequences, which are abundant throughout genomes; the *P. gingivalis* ATCC 33277 genome only has four NCBI annotated genes that lack a TA site, all of which are hypothetical proteins and are less than 40 amino acids in length [24-26]. No other constraints or preferences for Mariner transposon insertion have been identified. Recently, several investigators paired mutagenesis with mini-transposon derivatives of the Himar 1 Mariner transposon with massively-parallel sequencing technology in strategies variously named Tn-seq, IN-seq and HITs [27-30]. These strategies allow for quantitative assessment of individual mutants in a library by sequencing the transposon-genome junctions. Complex Mariner transposon libraries, in some cases saturating, have been used to define essential genes of several bacterial species including *Bacteroides thetaiotaomicron, Campylobacter jejuni*, *Haemophilus influenzae*, *Staphylococcus aureus* and *Streptococcus pneumoniae*[29-34]. Data from these studies have been collated into a Database of Essential Genes (DEG) [35-37]. Comparison of essential genes between bacterial species included in the DEG reveals that a large fraction of essential genes are species-specific. *P. gingivalis* essential genes cannot simply be inferred from studies in other bacteria, and such studies in *P. gingivalis* have not been performed to date, although a ‘core’ genome has been described by comparing ten strains by DNA/DNA-hybridization using microarray technology [38]. However, while there is likely to be overlap, a core genome does not equate with the set of genes essential for survival, and likely includes both essential and non-essential genes.

We have successfully adapted a Mariner-based transposon mutagenesis system to create highly-saturated mutant libraries in *P. gingivalis*. Here we describe our construction and analysis of this mutant library to identify essential genes in *P. gingivalis* and compare these genes to those identified in other bacteria.

## Results and discussion

### Generation of the mutant library

We generated transposon insertion libraries in *P. gingivalis* using a Himar 1 Mariner mini-transposon system created for use in *Bacteroides thetaiotaomicron*[30]. The *B. thetaiotaomicron* promoter of BT1331 that drives expression of *himar1c9a* transposase is recognized by *P. gingivalis*, allowing us to use the *B. thetaiotaomicron* plasmid vector pSAM_Bt with modifications in growth media and antibiotic selection conditions. This mini-transposon is constructed with two translational terminators downstream of the gene for antibiotic selection, thus eliminating read-through downstream from the insertion.

**Figure 1:**
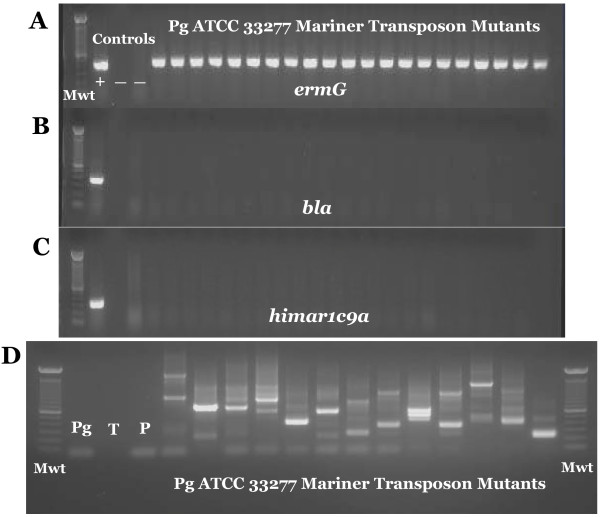
**Determination of proper transposon insertion.** Confirmation of transposon insertions was performed by PCR for presence of transposon (*ermG*) (**A**). “Mwt” = molecular weight marker, “+” = positive control (gDNA from *E. coli*/pSAM_Bt); “-“= a negative control (*P. gingivalis* ATCC 33277). All other lanes contain amplicons from PCR of individual colonies of transformed *P. gingivalis*. Panels (**B**) and (**C**) show PCR of the same samples using primers for the *bla* and *himar1c19a* genes respectively that are present in the plasmid, but which should be lost with proper insertion of the transposon. These three panels are a combination of separate gels; all which were run using identical PCR gDNA template for each of the separate reactions. (**D**) Nested semi-random PCR for individual mutant sequencing preparation. PCR from individual colonies was performed using primers to Mariner transposon and random primers ARB1 and ARB2 (Additional file 6: Table S6). Two rounds of nested PCR were performed. Negative controls of wild-type *P. gingivalis* strain ATCC 33277 (*Pg*), template only (T) and primer only (P) lanes precede thirteen individual mutants.

We performed mutagenesis using pSAM_Bt with *P. gingivalis* strain ATCC 33277. The 4.6 kb pSAM_Bt vector containing the Mariner mini-transposon cannot replicate in *P. gingivalis* and, in addition, the plasmid lacks sequence homology with the *P. gingivalis* genome. Therefore, after the plasmid enters *P. gingivalis* by transformation, transposition from the plasmid into the genome occurs without significant background insertion of the plasmid into the genome by illegitimate recombination. This system allows for single, stable transposition events since transposase activity is lost along with the plasmid. We collected 54,000 transposon insertion strains (individual colonies) from six separate transformation experiments. Variable colony sizes were observed among the mutants harvested and pooled following 14 days of growth. However, the majority of macroscopically visible colonies were similar in size to those of wild-type *P. gingivalis* strain ATCC 33277 after 14 days of growth. To confirm that the strains contained transposon insertions and not cryptic or full plasmid integrations, we performed PCR specific for the transposon (*ermG*) as well as for two portions of the vector backbone (*himar1c9a* and *bla*) (Figure 1A). Of 100 colonies that were screened, all showed positive PCR reactions for the transposon gene and negative reactions for the vector backbone, indicating ‘correct’ transposition. ‘Incorrect’ transpositions can include portions of the vector backbone inserting with the transposon, the vector being stably maintained within the bacterium extra-chromosomally or multiple insertions within the same genome; such transposition events were not detected in the subset of mutants tested (Figure 1). To determine whether the transposon inserted into different genes and not preferentially into genetic ‘hot-spots’, we performed nested semi-random PCR followed by sequencing which confirmed that insertions occurred in multiple locations across the genome (Figure 1D) [39]. This traditional sequencing method is effective for targeted sequencing a subset of mutants from the mutant library if massively-parallel high-throughput are neither desired nor necessary.

### Validation of Tn-seq of the *P. gingivalis* library

Having confirmed via nested semi-random PCR and subsequent sequencing that the libraries contained different transposon insertions scattered throughout the genome, we identified the location of each insertion in the library by Tn-seq analysis [27,28]. This method couples transposon mutagenesis with massively-parallel, next-generation sequencing technology to identify the location of each insertion and quantitate the relative abundance of each insertion mutant in the library.

Prior Tn-seq experiments using Mariner libraries have taken advantage of an engineered *Mme*I restriction site that cuts 18–20 base pairs away from the recognition site into the genomic DNA [27,30]. This method has been successfully employed to evaluate library sequences in a variety of settings, however, it suffers from a number of disadvantages including: 1) Yielding small sequencing reads limited to 16–18 nucleotides in length. 2) Requiring the use of a mutant transposon and hence existing transposition vectors must be mutated. 3) *MmeI* generates 2 base pair 3’ overhangs at adjacent sequences to which adapters are ligated. It is possible that the enzyme cleaves these adjacent sequences with varying efficiency. Furthermore, T4 DNA ligase is likely to join adapters to these varying overhangs with differential efficiency (for instance GG should be more efficient than AA). Such variations in efficiencies, if they exist, would lead to unequal representation of sequenced insertions. An alternate method for sequencing from junctions in transposon libraries involves the ligation of adapters to sequences near transposon junctions [29]. However, the method is labor intensive, requires gel purification of ligated products, and is prone to the unintended creation of inhibitory adapter dimers and other side products.

**Figure 2:**
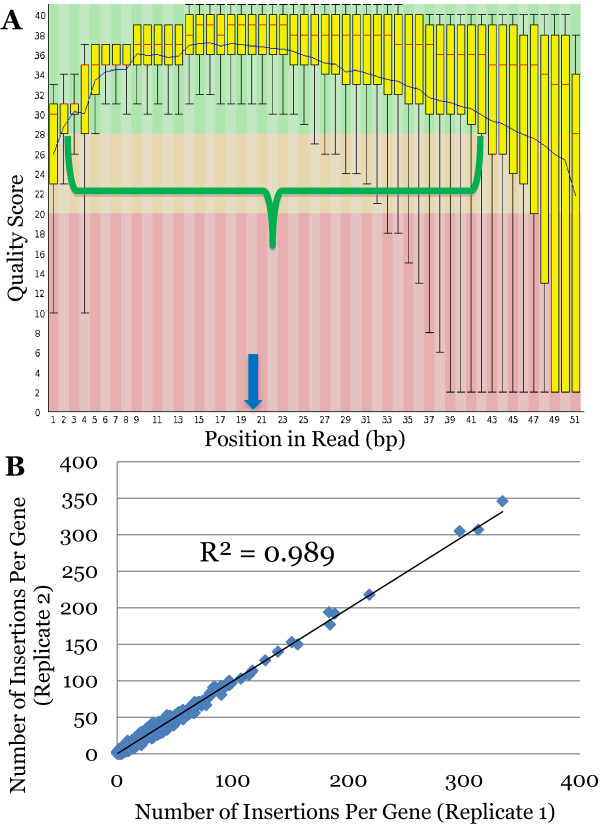
**Sequencing quality control and reproducibility.** Panel **A** shows quality scores of the Illumina sequencing reads for mapping. Fifty base pair single-end reads were obtained with ‘high’ quality out to ~42 base pairs and ‘good’ quality out to ~47 base pairs. The green background corresponds to high quality reads, the yellow background to intermediate quality reads and the red background to poor quality reads. Data shown are for the number of high, intermediate and low quality reads at a specific number of base pairs away from the transposon. The yellow bar encompasses the 25-75^th^ percentile and the red horizontal bar indicates the mean. The green bracket identifies the base pair position where high quality reads comprise the over 75% of the total reads. Blue arrow signifies where typical amount of sequencing that can be obtained when preparing DNA using the *MmeI* restriction site, demonstrating superior mapping and analysis ability of C-tailing method. No sequencing reads shorter than 20 bp were used for analyses. **B**) Replicates of the same library were sequenced in separate experiments. The graph compares the number of insertions per gene for technical replicates 1 and 2 of *P. gingivalis* strain ATCC 33277 Mariner mutant library and showed excellent correlation between the replicates (R^2^=0.9892). Median number of insertions when excluding genes containing zero is 9 while the mean is 17. Sixteen genes have 100 insertions or greater.

Here we report a new method, without the abovementioned disadvantages, for the construction of high-throughput sequencing libraries from transposon, retrovirus or repetitive element insertions sites in any genome. For details see the Materials and Methods section. In brief, genomic DNA containing the insertion element of interest is sheared, and then Terminal deoxynucleotidyl Transferase (TdT) is used to add an average of twenty deoxycytidine nucleotides to the 3’ ends of all molecules. Two rounds of PCR using a poly-C-specific and an insertion element-specific primer pair are then used to amplify one of the two insertion element-genomic DNA junctions and append all user-defined sequences needed for high-throughput sequencing and indexing. This new method does not require a ligation reaction, does not produce adapter dimers, does not require gel purification and is compatible with long sequencing reads the size of which is only limited by the length of library fragments and the sequencing technology. Here, in contrast to the 16–18 nucleotide reads obtained with the *MmeI* method we used 50 nucleotide reads allowing for significantly more effective and precise mapping of sequences to regions with nucleotide repeats as well as genes that contain nucleotide homology (Figure 2A). This is particularly important given that the current Illumina HiSeq2000 base-calling algorithm gives poor quality scores for the first few bases (Figure 2A).

Two replicate samples derived from the same master mutant library, but processed separately for sequencing, were compared. Sequencing revealed 35,937 and 35,732 distinct insertions (mutants) respectively (Figure 2B). Of the total insertions, 7,230 and 7,193 in the respective replicate runs were in putative intergenic regions. After quality filtering sequencing reads an average 6,310,573 reads could be attributed to an average of 35,835 insertions mapped to the genome. Of note, during multiplexed Illumina sequencing runs between 10–20 percent of sequencing reads are ‘thrown out’ during quality control analyses. This level of ‘discarded’ read data is seen by all groups performing permutations of Tn-seq, RNA-seq, ChIP-seq and other massively-parallel adapted methods. Sequencing data removed during our quality control analyses was within the 10–20 percent range previously noted. The number of insertions per gene and the number of reads per gene when comparing the technical replicates gave R^2^ values of 0.989 and 0.998 respectively (Figure 2B). The similarity between the two technical replicates demonstrates that aliquot production from the master library, processing of the samples as well as sequencing and analyses are highly reproducible.

### Identifying putative essential genes of *P. gingivalis* by Tn-seq

The genome of *P. gingivalis* strain ATCC 33277 comprises 2.35 Mb of chromosomal DNA and no plasmids. With a GC-content of 48.4%, there are 2,155 genes, 2,090 protein-coding sequences, 53 tRNA, and 12 rRNA [GenBank: AP009380.1] [9,40,41]. An important factor for Mariner transposition is that the genome contains 117,742 informative ‘TA’ sites, the only known specific motif ‘required’ for Mariner transposition. In previous studies and in agreement with our sequencing results, approximately 98% of Mariner insertions occur at TA sites (unpublished results).

The presence in our library of a mutant bacterium, in which a gene or intergenic region has been interrupted by insertion of the Mariner transposon, would indicate that it is unlikely that the gene or region is essential for growth *in vitro* on blood agar plates, provided that the insertion was likely to have inactivated the function. Insertions into the first or last five percent of a gene have a higher likelihood of generating a functional gene product relative to insertions in the middle portions of a gene, therefore these mutants were eliminated from consideration. In addition, we required a minimum of three sequencing ‘reads’ of the mutant locus be present in both technical replicates to exclude nonexistent insertions introduced by mapping of incorrectly sequenced reads, and lower rates may be due to mis-assignment by the reference assembly software. By these criteria, we determined that 1,639 genes are non-essential for growth *in vitro*. Sixteen of these genes contained 100 or greater insertions, notably the proteinases/adhesins *kgp* (310), *rgpA* (300), *rgpB* (152) and *hagA* (340) as well as the minor fibrilin *mfa1* and four of the twelve 23S rRNA genes. Eighty-eight genes contained 50 or more insertions and 837 contained 10 or more insertions, with a median number of 10 insertions per gene. There is a direct, but not completely exclusive, correlation between number of insertions and sequencing reads as 9 of the top 10 highest reads from the library belong to genes with more than 100 insertions; thus *kgp*, *rgpA*, *rgpB* and *mfa1* are in the top ten most-read genes. Nine hundred and twenty genes had transposon insertions in at least 25% of their reported TA sites, while a remaining 528 genes had insertions in at least 10% of their reported TA sites. The average number of TA sites per gene, when including all 2155 genes (CDS, tRNA and rRNA), is 55. A total of 87 genes were fully saturated with at least one mutant insertion into every available TA site in the gene. Full saturation results in a TA insertion ratio (actual number of different insertions into the TA sites of a gene divided by the theoretical maximum number of different insertions into the TA sites of a gene) of 1. A TA site to insertion ratio of greater than 1 demonstrates that at low frequency Mariner will insert into non-TA sites, most likely due to medium composition such as salt concentration that alleviate transposon specificity, local DNA structure, nucleotide composition and/or DNA-binding proteins. Of the 87 fully saturated genes having on average have 49 TA sites, 64 are present in multiple copies throughout the genome (Additional file 1: Table S1) and include rRNA genes, IS*Pg1*, IS*Pg3*, gingipains, and hypothetical proteins (Additional file 1: Table S1). All of the rRNA genes (12 in total) are located in spatially separated clusters of three and are fully saturated. Efforts are currently underway to determine whether there is conservation among non-TA insertion sites.

**Figure 3:**
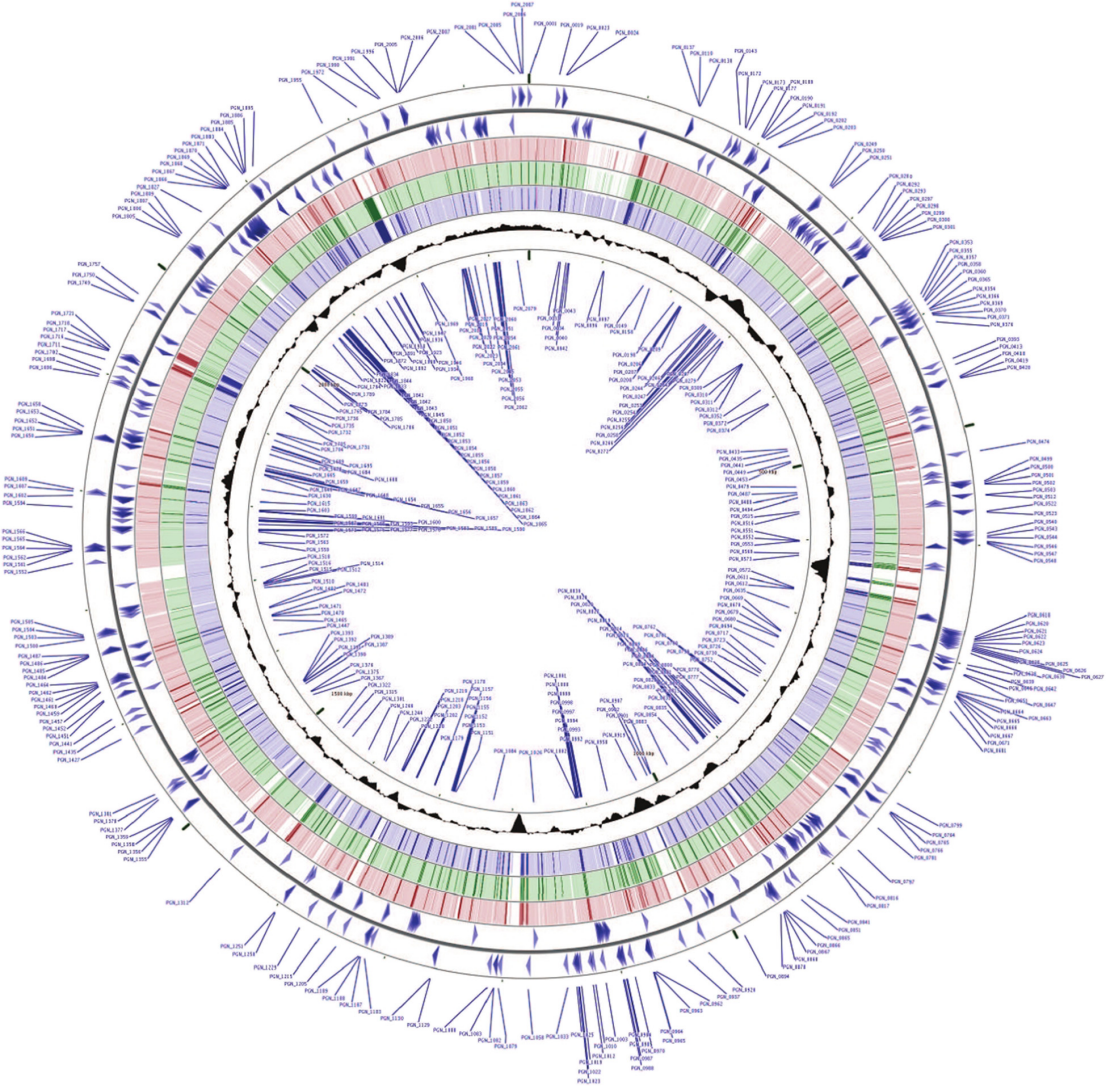
**Mapping of the *P. gingivalis *essential genes.** In blue in the outermost ring are shown the putative *P. gingivalis* essential genes identified by transposon mutagenesis of strain ATCC 33277. Blue arrows depict the orientation of the essential genes. Genetic loci for positive strand (outer set of arrows) are shown in the outermost circle and genetic loci for the negative strand are shown in the innermost ring. In red tick marks are coding sequences for *P. gingivalis* strain W83, in green are coding sequences for strain W50 and in blue are coding sequences for strain TDC60. Shaded black area represents GC-content for given regions. CGViewer (http://stothard.afns.ualberta.ca/cgview_server/) was utilized to visualize the entire circular genome of *P. gingivalis* strain ATCC 33277 with the putative essential genes labeled [52]. NCBI FASTA files of *P. gingivalis* strains W83, W50 and TDC60 were used for BLAST matching to the ATCC 33277 base genome.

For the remaining putative essential genes we applied the following rules to identify those most likely to be essential for *in vitro* survival. The rules are similar to those used in previous essential gene analyses of other bacteria and contend that [27,29,30,42]: 1) A gene must contain at least 10 TA sites. Genes with less sites could be under-inserted due to random chance. Of the 204 genes in the *P. gingivalis* ATCC 33277 genome with less than 10 TA sites, 189 (93%) are annotated as hypothetical. 2) Genes found to have an actual to theoretical insertion ratio of 50-fold or greater under-insertion were considered putatively essential (actual:theoretical ≤ 0.020). Applying these rules, out of a total 2,102 genes in the ATCC 33277 genome (all protein coding sequences and rRNA genes combined minus the 53 tRNAs), we identified 463 (22.0%) genes as putatively essential for *in vitro* survival (described below) (Figure 3) (Additional file 1: Table S1). Twenty-two percent of a 2.35 Mb genome containing 2,102 genes is within the range of essential genes determined by transposon mutagenesis, single gene deletions and *in silico* analyses of other bacterial genomes, as described below [29,30,32-34,42-51].

Prior to applying any cutoffs described above we found that 273 putative essential genes contained zero insertions. Given that these genes were a minimum of 200 base pairs in length and contained at least 10 TA sites the confidence level for concluding these as essential is high. Of the remaining 190 putative essential genes, 64 were found to have a ratio of between 0.001-0.010, 100-fold or less under-inserted, and 76 had a ratio between 0.010-0.020, 50-fold or less under-inserted. In most cases these genes had a single insertion over a gene length of 1.5-3.0 kb. Fifty genes had ratios between 0.020-0.050, however, these insertions were found to fall under the constraints outlined above and also met our qualifications for putative essentiality as well. Of note, of these 50 genes the majority (72%) have homology to genes of other bacteria identified in previous essential gene studies [35,37].

**Figure 4:**
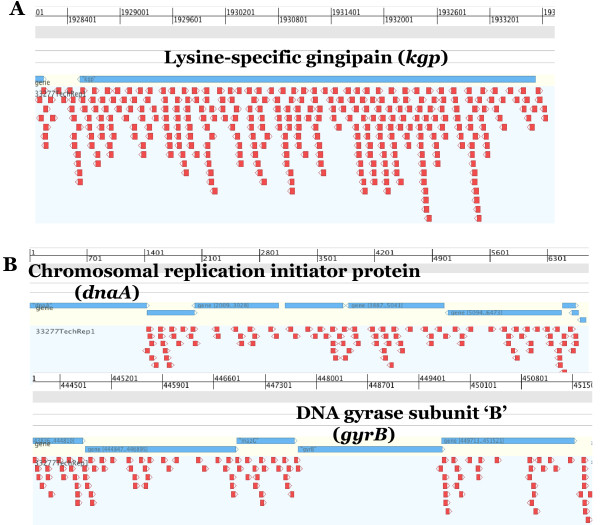
**Examples of transposon insertion distribution into highly-saturated and essential genes.** Panel **A** shows the insertion positions of the transposon for a very highly inserted gene, lysine gingipain, *kgp*. The blue bars represent the gene sequence. Each red arrow represents the location and orientation of a single insertion in the library. In panel **B**, we show two examples of essential genes, chromosomal replication initiator protein, *dnaA* and DNA gyrase subunit ‘B’, *gyrB*. As shown, there are numerous insertions in the flanking genes extending from the stop of *dnaA* and to the start and stop of *gyrB.*

**Figure 5:**
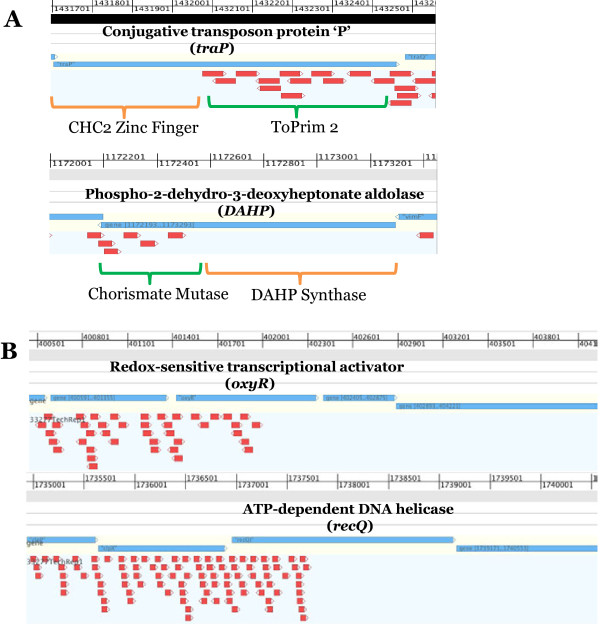
**Examples of transposon insertion distribution into genes that express proteins with essential and non-essential domains.** Panel **A** shows two examples of genes with multiple protein domains, one of which is essential and the other is not. In panel **B**, two non-essential genes, *oxyR* and *recQI*, are shown. Of note is the lack of transposon insertions into the latter regions of these genes. This would not be predicted based on the prevalence of TA sites in the non-inserted region of *oxyR* or *recQI*. GenomeView (http://genomeview.org/) was utilized for mapping and visualization of the transposon mutant insertion sequencing reads from Illumina sequencing to the *P. gingivalis* base genomes. BED file tracks created from Galaxy platform analyses were used for mapping insertions to a GenBank file base track.

In addition to identifying the essential nature of a gene, more detailed analysis, specifically mapping domains of proteins and intergenic regions, can provide valuable information about protein functional domains, promoter regions, mis-annotations, operon structure and regulatory RNAs (Figure 4/Figure 5). Simply mapping the insertions onto the genome to view saturation of specific genes provides a qualitative understanding of library complexity (Figure 4A). Annotations of genomes identify gene/coding-sequence start and stop codons, spatial relationships to other genes, operon structure, number of possible amino acids and amino acid composition. Such bioinformatic analyses are not perfect because they are based on coding-sequences from model organisms, e.g. *Escherichia coli,* and not adapted to less well-known bacterial species. Detailed insertion mapping allows for the determination of essential genes on a visual scale (Figure 4B). In addition, transposon mutagenesis mapping may reveal previously mis-annotated start and stop sites for genes as well as putative internal start sites, providing information on potential operon structure. Furthermore, essentiality of function domains can be determined (Figure 5A/5B). Although intergenic regions are far less abundant in prokaryotic genomes, mapping of insertions, or a lack thereof, to a specific intergenic region within the genome can provide insights on regulatory features within non-coding DNA sequences.

### Comparison of *P. gingivalis* essential genes to core genome and transcriptome

The core genome of *P. gingivalis* previously proposed by Brunner *et al*. was derived from hybridization analysis of 10 different strains to a DNA microarray of annotated genes from strain W83 [38]. Of note, any gene ‘missing’ from strain W83, even if present in all other strains of *P. gingivalis*, would be considered not part of the ‘core’. Since both strains W83 and ATCC 33277 are now fully sequenced, it is known that 8 genes in the *P. gingivalis* ATCC 33277 essential list are missing in W83. Five of these genes have been identified in a third sequenced and annotated strain, TDC60. There was nearly complete overlap between putative essential genes determined by Tn-seq with the *P. gingivalis* core genome, 434 of 463 (93.7%) putatively essential genes overlapped (Additional file 1: Table S1) [38]. Also, several gene probes were left out of the core genome analysis due to low hybridization signals or redundancy; two of these are identified as essential in our study. Nearly half (12 out of 31) of the essential genes not found in the *P. gingivalis* core genome had BLAST matches in the Database of Essential Genes (DEG) [35,37]. The remaining small difference may be explained by the hypothesis that certain essential genes are strain- and not species-specific, and thus may not be identified in a core genome analysis. In the circular genome representation of the base genome *P. gingivalis* ATCC 33277 (Figure 3) essential genes are depicted in arrows denoting directionality (blue) and homologous coding sequences are shown as tick marks in strains W83 (red), W50 (green) and TDC60 (blue). The map also shows that areas of genetic aberrance between *P. gingivalis* strains are areas devoid of essential genes (Figure 3). This would be hypothesized as essential genes should be conserved throughout a species unless duplication or gain-of-function mutation occur that can rescue the essential role of a give gene. As more *P. gingivalis* strain genomes are sequenced, bioinformatic analyses that provide mapped read-outs will delineate putative essential, core and accessory genetic regions, thus giving insight into strain-based differences within the species. Such differences may be useful to identify strain phylogeny and aid in clinical treatment regimens based on knowledge of genotype-to-phenotype virulence attributes (eg. antimicrobial resistance and gene transfer).

Chen *et al*. performed RNA-seq analysis of mRNA expression by *P. gingivalis* strain W83 from which 455 of the possible 463 ATCC 33277 essential genes were assayed [53]. This analysis demonstrated that 452 of 455 *P. gingivalis* ATCC 33277 essential genes were expressed during growth on blood agar medium (Additional file 1: Table S1). The 3 genes not expressed on blood agar plates as determined by RNA-seq are annotated as ‘hypothetical’ proteins. Transcriptome analyses were also performed on *P. gingivalis* grown on minimal (MIN), tryptic soy (TSB) and blood agar (BA) media, however, no essential genes were expressed solely on BA and not TSB or MIN despite some differences in levels of expression between the three media.

### Comparison of *P. gingivalis* essential genes with other essential gene analyses

Of the 463 putative essential genes in *P. gingivalis*, 364 (78.6%) have known essential gene homologues determined by BLASTP interrogation of the DEG (http://tubic.tju.edu.cn/deg/), version 6.8, updated on November 4, 2011 (Additional file 1: Table S1) [35,37]. The DEG curates a searchable list of “Essential genes [that] are those indispensable for the survival of an organism, and therefore are considered a foundation of life”. *P. gingivalis* essential genes were determined to have DEG homologues based strictly on BLASTP similarity. BLASTP similarities that resulted in e-values of 1x10^-8^ or less were considered matches. Homologies were found in at least one of the following species which had previously undergone essential gene studies: *Bacillus subtilis*, *B. thetaiotaomicron*, *E. coli*, *Francisella novicida*, *Haemophilus influenzae*, *Helicobacter pylori*, *Mycobacterium tuberculosis, Mycoplasma genitalium*, *Mycoplasma pulminous*, *Saccharomyces cerevisiae*, *Salmonella* Typhimurium*, Staphylococcus aureus*, *Streptococcus pneumoniae* and *Vibrio cholerae*[29,30,33,34,43,45-47,50,51,54-61]. For more than half of the 364 BLAST-matching essential genes there was homology within two or more species. In cases where only one other species contained a BLASTP match to a *P. gingivalis* essential gene it was most frequently to a gene in *B. thetaiotaomicron*, *H. influenzae* or *H. pylori*, which are the most closely related species to *P. gingivalis* both based on phylogeny and ecology.

The remaining 21.4% of putative essential genes that have no known homologue in the DEG may be essential in a species-specific or niche-specific manner. These 99 genes, many of which are functionally classified as containing known Pfam protein motifs, ‘conserved domains’ or ‘hypothetical’ proteins, may reveal important aspects related to metabolism and physiology of *Porphyromonas* species and closely related organisms. Of the 46 annotated as hypothetical proteins, 42 are among the 99 *P. gingivalis* essential genes not previously known to be essential from other studies.

Of the organisms for which an essential gene set has been identified, *H. influenzae*, *F. tularensis*, *Acinetobacter*, *M. tuberculosis*, *Salmonella* Typhimurium, *S. aureus* and *B. thetaiotaomicron* are the most relevant based on genome size, ecological niche and genetic relatedness to *P. gingivalis*. The determined essential genes of the above species were 1,657 genes with 462 essential (28%); 1,719 genes with 390 essential (23%); 3,307 genes with 499 essential (15%); 3,988 genes with 614 essential (16%); 4,314 genes with 353 essential (8%); 2,892 genes with 351 (12%); and 4,902 genes with 325 (6.6%), respectively [30,34,44,47,56,57,62].

*P. gingivalis* is a member of the *Bacteroidetes*, and before reclassification was known as *B. gingivalis*. There are no *Bacteroidetes* species or other anaerobes represented in the DEG, however, a putative list of *B. thetaiotaomicron* strain VPI-5482 essential genes is available from the supplemental material of Goodman *et al*. 2009 [30]. *B. thetaiotaomicron* strain *VPI*-5482 was originally isolated from human feces. The strain contains a 6.26 Mb chromosome and 0.03 Mb plasmid (NC_004663.1/NC_004703.1) with 4,864 genes (chromosome) and 38 (plasmid), 4,778 protein coding sequences (chromosome) and 38 (plasmid), 71 tRNA and 15 rRNA genes [63] [GenBank: AE015928.1 and AY171301.1]. In comparison, *P. gingivalis* ATCC 33277 has 43% (numerically) of protein coding sequences in a genome 37% of the size of that of *B. thetaiotaomicron* VPI-5482. It was estimated that *B. thetaiotaomicron* VPI-5482 contains 325 “candidate essential genes” [30]. Maintaining a larger genome and gene set provides more opportunities for functional redundancy and alternative pathways which can lead to a relatively smaller number of essential genes. Thus, 268 of 325 (82.5%) of *B. thetaiotaomicron* ‘essentials’ have BLAST homologues in *P. gingivalis* strain ATCC 33277 and of these, 78% (209 of the shared 268) are also essential in both organisms (Additional file 1: Table S1). Fifty-nine *B. thetaiotaomicron* BLAST matches are not essential in *P. gingivalis* and 57 have no BLAST match at all in the organism (Additional file 2: Table S2). A significant number of the shared essential genes (25 of the 209) are not characterized in the DEG (Additional file 3: Table S3) and of these 25 *Bacteroidetes*-specific essentials, three are annotated as permeases and two appear to be regulatory. Three essentials, PGN_1026, PGN_1481 and PGN_0249, are likely associated with capsular polysaccharide biosynthesis based on PGN_1026 and PGN_0249 being involved in the dolichol pathway and PGN_1481 functionally annotated as polysaccharide biosynthesis related. Parsing out essential genes of specific groups of species, in this case *Bacteroidetes* and/or anaerobes, can allow for specific drug targeting or directed nutrient supplementation.

In agreement with multiple previous studies on essential genes of bacteria, in *P. gingivalis* a significantly greater number of essential genes (248 or 53.6%) are found on the negative DNA strand, and 215 (46.4%) are found on the positive DNA strand (Additional file 1: Table S1) [64]. Similarly, there is a greater than expected proportion of enzymes, especially those within multiple functions or involved in multiple pathways, within the essential gene groups [65].

**Figure 6:**
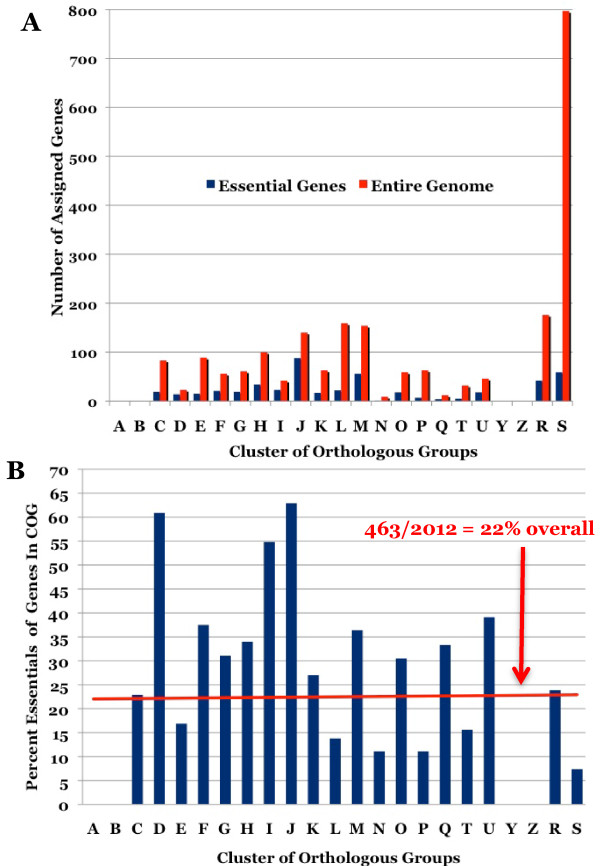
**Distribution of *P. gingivalis *ATCC 33277 essential genes by Cluster of Orthologous Groups (COG) classifications.A**) Number of genes within a COG category; essential genes in blue and entire genome in red. **B**) Percent of essential genes within a COG category from total number in genome; red line represents the 22% that 463/2102 of the percent essential over the entire genome. [A = RNA processing and modification, B = Chromatin structure and dynamics, C = Energy production and conversion, D = Cell cycle control, E = Amino acid metabolism and transport, F = Nucleotide metabolism and transport, G = Carbohydrate metabolism and transport, H = Coenzyme metabolism, I = Lipid metabolism, J = Translation, K = Transcription, L = Replication and repair, M = Cell wall/membrane/envelop biogenesis, N = Cell motility, O = Post-translational modification, protein turnover, chaperone functions, P = Inorganic ion transport and metabolism, Q = Secondary structure, T = Signal transduction, U = Intracellular trafficking and secretion, Y = Nuclear structure, Z = Cytoskeleton, R = General functional prediction only, S = Function unknown].

Using the Cluster of Orthologous Groups (COG) functional class designations (NCBI), we identified significant enrichment of essential genes within groups ‘D’ (cell cycle control/cell division), ‘I’ (lipid transport and metabolism) and ‘J’ (Translation/Ribosome); and a lack of enrichment was seen in ‘S’ (function unknown), ‘P’ (inorganic ion transport and metabolism) and ‘N’ (motility) (Figure 6A/6B) [66-68]. Enrichment (or lack thereof) of essential genes in these categories has been reported previously, however, essential gene enrichment in specific COG categories appears to be a species-specific characteristic.

Based on operon prediction and known essentials contained in the DEG it was determined that 25 of the 463 putative essential genes of *P. gingivalis* identified by Tn-seq may be the result of polar effects of the transposon insertion on downstream essential genes (Additional file 1: Table S1). Specifically, these 25 genes were identified as being upstream and potentially in an operon with one or more known essential genes, and additionally do not have BLAST matches in the DEG. Further study of each of these genes would be required to confirm their essentiality.

**Figure 7:**
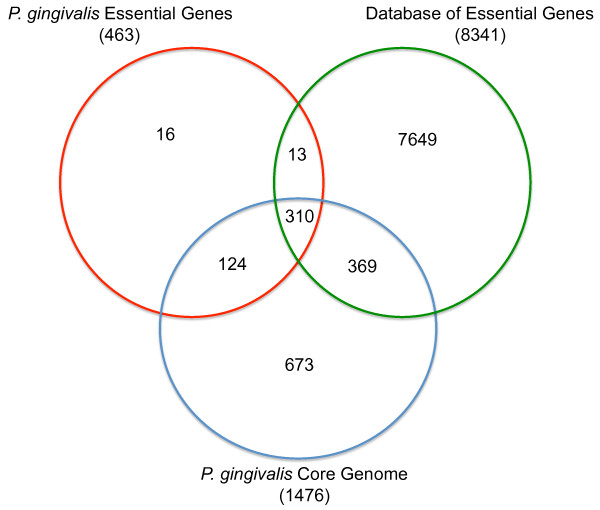
**Comparison of *P. gingivalis *essential genes, core genome and the entire DEG.** The red circle represents the distribution of the 463 *P. gingivalis* essential genes (determined using strain ATCC 33277), blue represents the distribution of the 1,476 gene *P. gingivalis* core genome and green represents the entirety of the 8,341 DEG curated genes. The innermost overlap shows genes found within all three datasets, while those found outside of all overlaps show genes specific to that individual dataset. Corresponding gene information can be found in Tables 1S and 3S.

Bringing the DEG and *P. gingivalis* core genome together in relation to *P. gingivalis* gene essentiality, we have determined that 369 genes within the core genome, ones not identified as essential in our study, have BLAST matches to genes within the DEG (Figure 7)(Additional file 4: Table S4). Within our mutant libraries we were able to identify transposon insertions into these genes such that they do not qualify as essential in *P. gingivalis*. Reasons for these genes being identified as essential in other species could be due to multiple variables such as *in vitro* selection media, species-specific essentiality, transposon type, library complexity, sequencing method, and criteria for essentiality. Such information gives importance to the distinction between a core gene set and an essential gene set as well as possible limitations of essential gene analyses based solely on *in silico* methodology.

### Characterization of *P. gingivalis* essential genes

Metabolic pathways that lack redundancy or have critical functions have been identified previously through essential gene studies. In our analysis of *P. gingivalis* we noted the presence of entire pathways as well as specific parts of pathways that are essential to *P. gingivalis* and to all other bacterial species. A subset of *P. gingivalis*-specific essential genes, possibly related to the ecological niche of the species, have also been identified (Additional file 1: Table S1). Of pathways involved in ribosome function we identified the *rpsA*, *rpmA*, *rplB* and *rimP* systems, which encode for 30S, 50S and maturation of ribosomes, respectively. The three major protein translation regulatory pathways of *infB*, *tsf* and *prfA,* as well as translational machinery pathway *rpoA* were found to be essential in our study. DNA replication, recombination and repair pathways of *dnaA* and *ruvA* as well as cell division pathways *mreB*, *parA* and *ftsA* were also found to be essential. Multiple pathways involved in LPS, CPS, fatty acid and murein biosynthesis, including *lpxA*, *rmlA*, *manA*, *fabD* and *murA* were also judged to be essential, as well as genes involved in secretion and chaperone pathways such as *secD*, *groES/EL* and *surA*. Pathways involving *nrfA*, *etfA*, *sufB*, *nadD* and *ribE* associated with oxidation-reduction reactions, were found to be essential in *P. gingivalis*, which is not surprising for an anaerobic bacterium. Major metabolic pathways *purA*, *pyrB*, *coaA*, *accB*, *pdxA*, *ispA*, *thiF*, *serA* and *dapA*, which encode nucleotide, amino acid and co-factor building blocks, respectively, were determined essential under our *in vitro* conditions. All of the aforementioned systems and pathways have previously been identified as essential for *in vitro* growth of other bacterial species, which is not unexpected given that replication, transcription, translation, cell division, membrane stability and central metabolism are key to survival [37].

Hypothetical genes are an often-overlooked group within any bacterial genome, including those of ‘model’ organisms. In our study we determined that approximately one-tenth of the essential genome of *P. gingivalis* encoded ‘hypothetical’ proteins, a few of which were homologous to other hypotheticals contained within the DEG. The majority of essential hypothetical genes are large and not within operons, suggesting that they encode functional proteins and are not essential due to a polar effect on a downstream essential gene. The finding that certain hypothetical proteins are essential will stimulate the search for protein motifs, structural bioinformatic and spatial organizational data and studies to define their function.

Although the notion that an essential gene within a given strain is likely to be essential in the species as a whole, intraspecies differences are known and often result in different phenotypes. For example, in strain ATCC 33277 we found no insertions into *ragA* and thus this gene was considered essential. Previous investigators also had difficulty making directed knockouts of *ragA* in strain W50; however, these investigators were successful in deleting *ragA* from strain WPH35. It is possible that *ragA* is only essential within specific strains and those strains in which it is non-essential compensate for loss of its function through the presence of other genes.

### Limitations of essential gene analysis

Limitations to essential gene studies should be addressed regardless of systems and methods utilized for their identification. First, several studies have relied exclusively on *in silico* bioinformatic analyses to determine essentiality. These analyses were based on programs designed to combine information from previous *in vitro* and *in vivo* mutagenesis studies with genome annotation and composition scripts without having carried out actual mutagenesis studies. Thus, any limitations of these experimental studies will be carried over into the new analyses and magnified by any inaccuracies of the program design itself. Second, in insertional mutagenesis methods to determine gene essentiality, genes may be misidentified as essential due to transposon insertion ‘cold-spots’. There is no ideal transposon identified as yet that completely lacks any nucleotide specificity and which can create completely random and saturating mutant libraries. Thus, no matter what type of transposon is used, Tn5, Tn7, Tn10, a cryptic construct or Mariner, all studies will have regions of the genome where fewer insertions occur. Third, genes that are actually non-essential but when mutated cause severe growth defects may be scored as essential due to practical limits to the depth of sequencing of transposon insertion junctions. These ‘sick’ mutants could potentially be represented at levels below 1000-fold a neutral mutant due to the number of replications it could go through prior to being pooled from mutagenesis plates into the library. Fourth, non-essential genes immediately upstream of and co-transcribed with essentials may be incorrectly scored as essential due to polarity of the transposon insertion. Last, practical limits to library complexity can result in some genes that fail to get disrupted by the transposon and so are misidentified as essential. This is particularly a problem for small genes or genes that are within cold spots for the transposon. Several studies, based mostly on the genome size of the species under investigation and the type of transposon, have attained different levels of saturation prior to analyses for essential genes. The possible limitation of our library when combining the type of transposon and library complexity relates to genes that contain less than 10 TA sites in their coding-sequences. Of the 204 genes with fewer than 10 TA sites, 60 could potentially be scored as essential based on having zero insertions, but do not qualify, given our stringent criteria (Additional file 5: Table S5). Adding confidence to the notion that many of these are non-essential is that 24 of the 60 genes encode proteins of less than 35 amino acids in length. Since these are all characterized as ‘hypothetical’ and are rather short to encode functional proteins, we believe that some of these may simply be artifacts of annotation programs and thus not true protein-coding genes.

Even complete gene deletion, non-transposon based studies of essential genes have limitations. The Keio collection of single and double gene deletions in *Escherichia coli* is considered the most comprehensive essential gene study to date [43,69]. Genes that could not be deleted were scored as essential, however, failure to delete a gene is not a guarantee of essentiality and there a are few genes identified as essential in the Keio collection that were successfully deleted by other labs. Furthermore, a handful of genes labeled non-essential were actually essential. The Keio deletions of those genes have second site suppressor mutations that compensate for the loss of the essential gene.

The best understanding of essential genes is likely to come from combining different modalities to confirm their essential nature and comparison of these databases both within and between species.

## Conclusions

We have described a method for performing Tn-seq with *P. gingivalis* using a Mariner mini-transposon and Illumina platform next-generation sequencing. We have also invented a new method for creating sequencing libraries from bacterial transposon libraries that has many advantages compared to previous methodologies. Using that method we identified specific mutants quantitatively in a highly reproducible manner using massively-parallel sequencing techniques. We used a near saturating insertion library generated in *P. gingivalis* strain ATCC 33277 to define the set of genes essential for growth on blood agar. Both the availability of a *P. gingivalis* mutant library and the ability to screen quantitatively are a marked advance in genetic and molecular tools for future studies of *P. gingivalis* biology and pathogenesis. By applying different selective pressures to the library, it is now possible to identify genes critical for survival and growth under different conditions. Due to the quantitative nature of results provided by Tn-seq, both positive and negative gene effects, including partial phenotypes, can now be readily identified.

## Methods

### Bacterial strains and plasmids

*P. gingivalis* ATCC 33277 (RefSeq NC_010729.1) was obtained from the ATCC. *E. coli* S17-1 λ*pir* and plasmids pSAM, containing *bla*, and pSAM_Bt, containing *bla*, *ermG* and *himar1c9a* genes were obtained from Dr. Andrew Goodman.

### Media and culture conditions

*Porphyromonas gingivalis* strain ATCC 33277 was grown and maintained at 37°C under anaerobic conditions using the GasPak™ EZ Anaerobe Pouch System (BD Biosciences). Blood agar plates (BAPHK) containing trypticase soy agar supplemented with defibrinated sheep’s blood (5% vol/vol), hemin (5 μg/ml), and menadione (0.5 μg/ml) as well as brain-heart infusion broth (BHIHKS_bc_S_tg_C) containing brain-heart infusion, yeast extract (1 mg/ml), hemin (5 μg/ml), and menadione (0.5 μg/ml), sodium bicarbonate (1 mg/ml), sodium thioglycolate (0.25 mg/ml), and cysteine (0.5 mg/ml) were used for solid and liquid culture of *P. gingivalis,* respectively. Gentamicin (25–50 mg/ml) and erythromycin (2–10 mg/ml) were used when appropriate for prevention of contamination as well as isolation and maintenance of *P. gingivalis* mutants.

*Escherichia coli* strain S17-1 λpir/pSAM_Bt was grown at 37°C under aerobic conditions in Luria broth base (LB) and Luria agar (BD Biosciences). Carbenicillin (50 μg/ml) was added for plasmid maintenance and prevention of contamination. *E. coli* S17-1 λpir contains the *pir* gene and has chromosomally integrated conjugational transfer functions (RP4/RK6) such that bi-parental mating can take place in lieu of tri-parental mating using helper strains.

### Transposon mutagenesis

*P. gingivalis* Mariner-based transposon mutagenesis was carried out as follows. Wild-type *P. gingivalis (*strain ATCC 33277) was inoculated into brain-heart infusion broth (BHIHKS_bc_S_tg_C) without antibiotics. Broth cultures were grown to optical densities (OD_600_) between 0.50 and 1.00. *Escherichia coli* strain S17-1 λ*pir* containing the pSAM_Bt plasmid was grown to optical densities OD 0.50 - 1.00. Broth cultures were set up such that between a 5:1 and 10:1 ratio of *P. gingivalis* (recipient) to *E. coli* (donor) was achieved. Although *P. gingivalis* is categorized as an obligate anaerobe it is able to survive without significant CFU loss (less than a log_10_) for up to 6 hours under aerobic conditions when incubated alone on BAPHK at 37°C.

The *E. coli* donor strain carrying the Mariner transposon on a suicide plasmid vector was conjugated with wild-type *P. gingivalis* using a bi-parental procedure where the *E. coli* donor strain and *P. gingivalis* recipient strain are cultured together on an agar plate to allow for plasmid transfer. Conjugation was carried out aerobically at 37°C for 5 hr. As *P. gingivalis* is naturally resistant to gentamicin, this antibiotic was used for selection against the donor *E. coli* following the conjugation. The transposon contains an erythromycin resistance gene (*ermG*) used to select for *P. gingivalis* transposon insertion mutants.

Individual *P. gingivalis* colonies were tested using PCR to detect the presence of the transposon as well as vector backbone components (*bla* and *himar1C9a* transposase). These tests indicated that few to no mutants contained incorrect/unwanted transpositions; vector insertions containing additional portions of the transposon-containing plasmid. Nested semi-random PCR and direct sequencing of the transposon-chromosome junction of individual mutants determined that the transpositions appear randomly distributed throughout the *P. gingivalis* chromosome.

### PCR

Mutant colonies were initially isolated directly from transposition plates (post-conjugation) and three times to ensure purity were sub-cultured under antibiotic selection on blood agar plates anaerobically. Genomic DNA was prepared using a DNeasy (Qiagen) kit as per the manufacturer’s instructions. PCR was performed using primers to detect the presence of *ermG*, *bla* (amp^R^) and *himar1C9a* genes as detailed in Additional file 6: Table S6.

PCR using semi-random priming was performed in order to determine the chromosomal location of the transposon insertion. Nested PCR primers directed to the pSAM_Bt vector (the remaining flanking regions of the transposon) were used in sequential PCR reactions and the final sequencing reaction. Primers are detailed in Additional file 6: Table S6.

Sequencing of the PCR amplicons was carried out at Tufts University Core Facilities using ABI 3130XL DNA sequencers.

### Construction and sequencing of libraries

Genomic DNA eluted in 100 μL elution buffer (Qiagen) was placed in a 2 mL microfuge tube and sheared for 2 minutes (10 sec on and 5 sec off duty cycle, 100% intensity) using a high intensity cup horn that was cooled by a circulating bath (4°C) and was attached to a Branson 450 sonifier. C-tails were then added to 1 μg of sheared DNA in a 20 μL reaction that contained 0.5 μL TdT enzyme (Promega), 4 μL 5x TdT reaction buffer (Promega), 475 μM dCTP and 25 μM dideoxy CTP. The dideoxy CTP functions as a chain terminator to limit the length of the poly-C tails. Following a 1-hour incubation at 37°C and a 20 minute heat-inactivation step at 75°C, dideoxy CTP and other small molecules were removed using a Performa gel filtration cartridge (Edge Biosystems). Transposon containing fragments were then amplified in a 50 μL PCR reaction that contained 5 μL C-tailed template, 600 nM C tail-specific primer (olj376 5^′^ GTGACTGGAGTTCAGACGTGTGCTCTTCCGATCTGGGGGGGGGGGGGGGG 3^′^), 600 nM transposon-specificprimer (pSAM1 5^′^ CCTGACGGATGGCCTTTTTGCGTTTCTACC 3^′^), 400 μM dNTPs, 5 μL 10x buffer, and 1 μL Easy-A DNA polymerase mix (Agilent). Sandwiched by an initial incubation at 95°C for 120 sec and a final extension of 120 sec at 72°C, 24 cycles were completed using 30 sec denaturation steps at 95°C, 30 sec annealing steps at 60°C, and 120 sec extension steps at 72°C. A second PCR reaction was then used to amplify the exact transposon-genomic DNA junction and add additional sequences needed for Illumina sequencing and indexing. This 50 μL reaction contained 1 μL of template from PCR #1, 600 nM transposon end-specific primer (pSAM2 5^′^ AATGATACGGCGACCACCGAGATCTACACTCTTTGACCGGGGACTTATCATCCAACCTGTTA 3^′^), 600 nM indexing primer (5^′^ CAAGCAGAAGACGGCATACGAGATNNNNNNGTGACTGGAGTTCAGACGTGTGCTCTTCCGATCT 3^′^, where NNNNNN represents the reverse complement of the index and varied with each sample), 400 μM dNTPs, 5 μL 10x buffer, and 1 μL easy-A DNA polymerase mix (Agilent). Sandwiched by an initial incubation at 95°C for 120 sec and a final extension of 120 sec at 72°C, 12 cycles were completed using 30 sec denaturation steps at 95°C, 30 sec annealing steps at 60°C, and 120 sec extension steps at 72°C. Libraries were then pooled and run for 51 cycles in a single end sequencing reaction on a single lane of an Illumina Genome Analyzer II (Tufts University) using the custom sequencing primer pSAM3 (5^′^ ACACTCTTTGACCGGGGACTTATCATCCAACCTGTTA 3^′^) and the standard Illumina index sequencing primer.

### Data analysis

All read mapping and primary data analysis was done on the Tufts University Galaxy server. Approximately seven-percent of all sequencing reads contained multiple ‘C’ nucleotides at their 3’ end as a consequence of the C-tailing reaction. These C-tails were removed using the “clip adapter sequences script” with the 3’ adapter set to CCCCCCCCCCCCCCCCCCCCCCCCCC and the minimum read length set to 26. The resulting clipped reads were aligned to the *P. gingivalis* strain ATCC 33277 and W83 reference genomes, accession numbers AP009380.1 and AE015924.1 respectively, using Bowtie with its default settings. The resulting bowtie output file was then used as input for a custom script, “hopcount”. Hopcount tabulates the number of times individual insertion sites in the genome were re-sequenced. An Excel spreadsheet file is generated that indicates, for each insertion site, its position in the genome, gene locus to which that position maps, the strand (positive vs. negative) associated with the site as well as the frequency of its reads. Hopcount output was used to estimate the complexity of transposon libraries and to compare the fate of specific insertions sites in input and output samples. It was also used as input for a second custom script, “aggregate hop table”. The output of this script is an excel file in which all transposon insertion sites are tabulated by their collective frequency in each annotated gene of the genome. For each gene, the number of unique insertions sites observed, absolute count of sites in the positive strand, in negative strand and in both strands is recorded. Also recorded is the normalized value dvalgenome, which is an indication of whether the number of insertions observed in that gene is above or below the expected frequency. Dvalgenome equals the observed number of insertions in a gene / predicted number of insertions for that gene and the predicted number of insertions (size of that gene in base pairs divided by size of genome in base pairs) multiplied by (total number of insertions counted).

### Bioinformatics resource for oral pathogens

Microbial Transcriptome Database, MTD (http://bioinformatics.forsyth.org/mtd/) maintained by the Forsyth Institute in Cambridge, MA, USA, was utilized for comparing the putative essential genes of *P. gingivalis* ATCC 33277 to that of the RNA-seq transcriptome information detailing gene expression when grown on blood agar medium (from strain W83) [70].

*P. gingivalis* essential genes were determined to have DEG homologues based strictly on BLASTP similarity. BLAST similarities at protein-protein level that resulted in e-values of 1x10^-8^ or less were considered matches. Pfam (http://pfam.sanger.ac.uk/), Welcome Trust Sanger Institute, Prosite (http://prosite.expasy.org/), Swiss Institute of Bioinformatics and Interproscan (http://www.ebi.ac.uk/Tools/pfa/iprscan/), European Bioinformatics Institute, protein information databases/platforms were utilized to query all *P. gingivalis* essential genes for functional motifs (including signal sequences) and post-translational/co-translational modification sites [71-73]. In cases where genes were previously described as un-annotated hypothetical proteins but were now found to have functional motifs they have added them to the analyses and lists. National Center for Biotechnology Information, NCBI (http://www.ncbi.nlm.nih.gov/), National Institutes of Health, USA was used for gathering genome information on *Porphyromonas gingivalis* stains ATCC 33277 and W83. The Kyoto Encyclopedia of Genes and Genomes, KEGG (http://www.genome.jp/kegg/), database contains genetic information on all three of the sequenced and annotated strains of *P. gingivalis* (ATCC 33277, W83 and TDC60). The entry number for *P. gingivalis* 33277 is T00714, which is the reference genome used to this study. All *P. gingivalis* ‘essential’ genes were examined for KEGG-described functional characterizations through the T00714 KEGG gene list. In cases where genes were previously described as un-annotated hypothetical proteins but were now found to have functional motifs they have added them to the analyses and lists. Bioinformatics Resource for Oral Pathogens, BROP (http://www.brop.org/) maintained by the Forsyth Institute in Cambridge, MA, USA, was utilized for *P. gingivalis* genome annotation, comparison between annotations by NCBI, BROP, TIGR and Los Alamos National Laboratories, operon structure analysis and BLAST (Basic Local Alignment Search Tool) of nucleotide and protein sequences between oral bacteria [70].

## Abbreviations

Pg: *Porphyromonas gingivalis*; Bt: *Bacteroides thetaiotaomicron*; PCR: Polymerase Chain Reaction; DEG: Database of Essential Genes; BHI: Brain-Heart Infusion; BAP: Blood Agar Plate; BROP: Bioinformatics Resource Oral Pathogens; MTD: Microbial Transcriptome Database; BLAST: Basic Local Alignment Search Tool; KEGG: Kyoto Encyclopedia of Genes and Genomes.

## Competing interests

AC and DWL have a patent application filed on the method for constructing DNA libraries for sequencing. The authors declare no other competing interests.

## Authors’ contributions

BAK conceived of the study, participated in its design and coordination, carried out molecular genetics, carried out bioinformatic analyses and drafted the manuscript. ELT participated in study design and coordination and drafted the manuscript. LTH conceived of the study, participated in its design and coordination and drafted the manuscript. DWL participated in study design and coordination and drafted the manuscript. AC participated in study design and coordination and drafted the manuscript. MJD participated in study design and coordination and drafted the manuscript. All authors read and approved the final manuscript.

## Supplementary Material

Additional file 1 **Table S1.***P. gingivalis* strain ATCC 33277 essential gene list; functional characterization and bioinformatic analyses. Green highlight denotes being found in the DEG as essential in other genomes, blue highlight denotes *Bacteroides thetaiotaomicron* (Goodman *et al*. Cell Host & Microbe 2009) but NOT DEG essential gene match and orange highlight denotes being part of *P. gingivalis* core genome (Brunner *et al*. BMC Microbiology 2010). (XLS 133 kb) Click here for file

Additional file 2 **Table S2.** Shared genes between *P. gingivalis* strain ATCC 33277 and *B. thetaiotaomicron* that are only essential in *B. thetaiotaomicron*. (XLSX 11 kb) Click here for file

Additional file 3 **Table S3.** Details of *P. gingivalis* strain ATCC 33277 essential genes shared only with *B. thetaiotaomicron*. (XLS 24 kb) Click here for file

Additional file 4 **Table S4.***P. gingivalis* core genome in relation to gene essentiality. The 1476 genes that comprise the *P. gingivalis* core genome are listed in order of their TIGR gene identification number (Brunner *et al*. BMC Microbiology 2010). Genes with their TIGR functional characterizations highlighted in green are *P. gingivalis* essential gene homologues in strain W83, while those highlighted in blue are non-essential *P. gingivalis* core genes that have BLAST matches within the DEG. BLAST matches were determined as having protein-protein similarity of e-values 1x10^-8^ or less. Black lettering within brackets describing “only in” denote what species in the DEG a core gene had similarity to if there was only one. Red lettering within brackets denotes BROP annotation to genes when differing from TIGR annotations. (DOC 858 kb) Click here for file

Additional file 5 **Table S5.***P. gingivalis* strain ATCC 33277 genes without insertions that are excluded from consideration as essential genes based on total TA site number. (XLS 24 kb) Click here for file

Additional file 6 **Table S6.**Primer sequences for vector-part PCR, nested semi-random PCR sequencing and Illumina sequencing Click here for file
