# Supplementary material for: Identification of essential genes of the periodontal pathogen Porphyromonas gingivalis
Source: BMC Genomics. 2012 Oct 31;13:578. doi: 10.1186/1471-2164-13-578 (PMC3547785; doi:10.1186/1471-2164-13-578)
Supplement: Additional file 4 — Table S4.P. gingivalis core genome in relation to gene essentiality. The 1476 genes that comprise the P. gingivalis core genome are listed in order of their TIGR gene identification number (Brunner et al. BMC Microbiology 2010). Genes with their TIGR functional characterizations highlighted in green are P. gingivalis essential gene homologues in strain W83, while those highlighted in blue are non-essential P. gingivalis core genes that have BLAST matches within the DEG. BLAST matches were determined as having protein-protein similarity of e-values 1x10^-8^ or less. Black lettering within brackets describing “only in” denote what species in the DEG a core gene had similarity to if there was only one. Red lettering within brackets denotes BROP annotation to genes when differing from TIGR annotations. (DOC 858 kb) [file 1471-2164-13-578-S4.doc]

| **GeneID** | **Functional Characterization** |
| --- | --- |
| *PG0001* | chromosomal replication initiator protein DnaA |
| *PG0002* | hexapeptide transferase family protein |
| *PG0003* | membrane protein, putative |
| *PG0004* | transcriptional regulator, Sir2 family |
| *PG0005* | conserved hypothetical protein |
| *PG0006* | MATE efflux family protein |
| *PG0007* | hypothetical protein |
| *PG0010* | ATP-dependent Clp protease, ATP-binding subunit ClpC |
| *PG0011* | glycosyl hydrolase, family 3 |
| *PG0012* | L-threonine-O-3-phosphate decarboxylase, putative |
| *PG0013* | conserved hypothetical protein |
| *PG0014* | ISPg2, transposase, truncation |
| *PG0016* | sigma-54 dependent DNA-binding response regulator |
| *PG0017* | sensor histidine kinase |
| *PG0018* | hypothetical protein |
| *PG0020* | transcriptional regulator, MarR family |
| *PG0021* | TIM-barrel protein, putative, NifR3 family |
| *PG0022* | sulfate permease family protein |
| *PG0024* | DNA-binding protein, putative |
| *PG0025* | fumarylacetoacetate hydrolase family protein |
| *PG0026* | hypothetical protein [Por secretion system protein porU] |
| *PG0027* | hypothetical protein [Por secretion system protein porV] |
| *PG0028* | 2C-methyl-D-erythritol 2,4-cyclodiphosphate synthase |
| *PG0029* | transposase, truncation-RNA methyltransferase, TrmH family, truncation |
| *PG0030* | cytidine deaminase |
| *PG0032* | beta-mannosidase, putative |
| *PG0033* | RmuC domain protein |
| *PG0034* | Thioredoxin |
| *PG0035* | DNA polymerase III, alpha subunit |
| *PG0037* | ribosomal protein L19 |
| *PG0039* | hypothetical protein |
| *PG0042* | serine hydroxymethyltransferase [glyA] |
| *PG0043* | beta-hexosaminidase |
| *PG0045* | heat shock protein HtpG |
| *PG0046* | phosphatidate cytidylyltransferase |
| *PG0047* | cell division protein FtsH, putative |
| *PG0048* | conserved hypothetical protein TIGR00092 |
| *PG0049* | hypothetical protein |
| *PG0052* | sensor histidine kinase |
| *PG0053* | hypothetical protein |
| *PG0054* | single-stranded-DNA-specific exonuclease RecJ |
| *PG0055* | conserved domain protein |
| *PG0056* | hypothetical protein |
| *PG0057* | nicotinate phosphoribosyltransferase |
| *PG0058* | nicotinate (nicotinamide) nucleotide adenylyltransferase |
| *PG0059* | hypothetical protein |
| *PG0060* | hypothetical protein |
| *PG0061* | yngK protein |
| *PG0062* | TPR domain protein |
| *PG0063* | outer membrane efflux protein |
| *PG0064* | heavy metal efflux pump, CzcA family [only in *Salmonella*] |
| *PG0065* | efflux transporter, RND family, MFP subunit |
| *PG0066* | hypothetical protein |
| *PG0068* | hypothetical protein |
| *PG0069* | conserved hypothetical protein |
| *PG0070* | acyl-(acyl-carrier-protein)-UDP-N-acetylglucosamine acyltransferase |
| *PG0071* | UDP-3-O-acyl-GlcNAc deacetylase-beta-hydroxyacyl-[acyl carrier protein] dehydratase FabZ |
| *PG0072* | UDP-3-O-[3-hydroxymyristoyl] glucosamine N-acyltransferase |
| *PG0073* | orotidine 5-monophosphate decarboxylase |
| *PG0074* | peptide chain release factor 1 |
| *PG0075* | phosphoribosylformylglycinamidine cyclo-ligase, putative |
| *PG0076* | N-acetylmuramoyl-L-alanine amidase, family 4 |
| *PG0080* | hypothetical protein |
| *PG0081* | hypothetical protein |
| *PG0082* | hypothetical protein |
| *PG0083* | hypothetical protein |
| *PG0084* | L-serine dehydratase, iron-sulfur-dependent, single chain form [only in *E. coli*] |
| *PG0085* | alpha-galactosidase |
| *PG0086* | ATP-dependent RNA helicase, DEAD-DEAH box family |
| *PG0087* | SIS domain protein |
| *PG0088* | peptidase, M16 family |
| *PG0090* | Dps family protein |
| *PG0091* | transporter, putative |
| *PG0092* | transporter, putative |
| *PG0094* | outer membrane efflux protein, putative |
| *PG0095* | DNA mismatch repair protein MutS |
| *PG0099* | phenylalanyl-tRNA synthetase, beta subunit |
| *PG0104* | DNA topoisomerase III |
| *PG0106* | glycosyl transferase, group 4 family protein |
| *PG0108* | UDP-N-acetyl-D-mannosaminuronic acid dehydrogenase |
| *PG0121* | DNA-binding protein HU |
| *PG0123* | hypothetical protein |
| *PG0124* | conserved hypothetical protein [only in *S. aureus*] |
| *PG0126* | type I phosphodiesterase-nucleotide pyrophosphatase family protein |
| *PG0127* | Ferrochelatase [hemH] |
| *PG0129* | mannosyltransferase |
| *PG0130* | phosphoglycerate mutase [gpmA] |
| *PG0132* | hypothetical protein |
| *PG0133* | hypothetical protein [polysaccharide biosynthesis protein] |
| *PG0134* | magnesium transporter |
| *PG0135* | dimethyladenosine transferase |
| *PG0136* | hypothetical protein |
| *PG0137* | aminoacyl-histidine dipeptidase |
| *PG0138* | malonyl CoA-acyl carrier protein transacylase |
| *PG0139* | membrane-bound lytic murein transglycosylase D, putative |
| *PG0140* | hypothetical protein |
| *PG0141* | spoOJ protein |
| *PG0142* | SpoOJ regulator protein |
| *PG0143* | hydrolase, carbon-nitrogen family |
| *PG0144* | conserved hypothetical protein [only in *Caulobacter crescentus*] |
| *PG0146* | hypothetical protein |
| *PG0147* | hypothetical protein [lipoprotein] |
| *PG0148* | sigma-54-dependent transcriptional regulator |
| *PG0149* | conserved domain protein |
| *PG0150* | conserved hypothetical protein TIGR01125 [rimO] |
| *PG0151* | signal recognition particle-docking protein FtsY |
| *PG0152* | carboxynorspermidine decarboxylase |
| *PG0153* | aspartyl-tRNA synthetase |
| *PG0155* | riboflavin biosynthesis protein RibD |
| *PG0156* | modification methylase, HemK family |
| *PG0157* | regulatory protein RecX |
| *PG0158* | competence protein F-related protein |
| *PG0159* | endopeptidase PepO |
| *PG0160* | conserved domain protein |
| *PG0161* | hypothetical protein |
| *PG0162* | RNA polymerase sigma-70 factor, ECF subfamily |
| *PG0163* | Phosphofructokinase[pfkA] |
| *PG0164* | conserved hypothetical protein |
| *PG0165* | heat shock protein 15 |
| *PG0166* | peptidyl-tRNA hydrolase |
| *PG0167* | ribosomal protein L25 |
| *PG0170* | methionyl-tRNA synthetase |
| *PG0171* | 5-nucleotidase family protein [only in *Haemophilus influenzae*] |
| *PG0172* | exonuclease |
| *PG0173* | transcriptional regulator, putative |
| *PG0188* | lipoprotein, putative |
| *PG0189* | hypothetical protein |
| *PG0190* | undecaprenyl diphosphate synthase |
| *PG0191* | outer membrane protein, putative |
| *PG0192* | cationic outer membrane protein OmpH |
| *PG0193* | cationic outer membrane protein OmpH |
| *PG0195* | rubrerythrin |
| *PG0196* | peptidase, M16 family [pqqL] |
| *PG0199* | TatD family protein |
| *PG0200* | conserved hypothetical protein TIGR00278 |
| *PG0201* | ribonuclease P protein component |
| *PG0202* | uroporphyrinogen-III synthase HemD, putative |
| *PG0203* | hypothetical protein |
| *PG0205* | peptide chain release factor 3 |
| *PG0209* | formate-nitrite transporter [only in *Haemophilus influenzae*] |
| *PG0210* | precorrin-6x reductase-cobalamin biosynthetic protein CbiD |
| *PG0211* | cobalamin biosynthesis protein CbiG-precorrin-4 C11-methyltransferase [only in *Acinetobacter baylyi*] |
| *PG0213* | precorrin-3 methylase-precorrin-8X methylmutase |
| *PG0214* | RNA polymerase sigma-70 factor, ECF subfamily |
| *PG0215* | hypothetical protein |
| *PG0216* | hypothetical protein |
| *PG0217* | hypothetical protein |
| *PG0218* | hypothetical protein |
| *PG0223* | exonuclease |
| *PG0224* | conserved hypothetical protein |
| *PG0226* | transglutaminase-related protein |
| *PG0227* | DNA repair protein RadA [only in *Haemophilus influenzae*] |
| *PG0228* | DdaH family protein |
| *PG0229* | hypothetical protein |
| *PG0230* | transaldolase TalC, putative [only in *Caulobacter crescentus*] |
| *PG0231* | conserved hypothetical protein |
| *PG0232* | zinc carboxypeptidase, putative |
| *PG0234* | immunoreactive 23 kDa antigen PG66 |
| *PG0235* | carboxyl-terminal protease [only in *Salmonella*] |
| *PG0236* | hypothetical protein |
| *PG0237* | uracil-DNA glycosylase |
| *PG0240* | hydrolase, haloacid dehalogenase-like family |
| *PG0241* | lipoprotein, putative |
| *PG0242* | conserved hypothetical protein TIGR00096 [methyltransferase] |
| *PG0243* | hypothetical protein |
| *PG0245* | universal stress protein family |
| *PG0246* | hypothetical protein |
| *PG0248* | translation initation factor SUI1, putative [only in *Vibrio cholerae*] |
| *PG0249* | oxaloacetate decarboxylase, putative [only in *Mycobacterium tuberculosis*] |
| *PG0253* | conserved hypothetical protein [ribosome maturation factor rimP] |
| *PG0254* | N utilization substance protein A, putative [nusA] |
| *PG0255* | translation initiation factor IF-2 |
| *PG0256* | CvpA family protein |
| *PG0257* | conserved hypothetical protein [cysteine disulfurase sufB] |
| *PG0258* | ABC transporter, ATP-binding protein |
| *PG0259* | conserved hypothetical protein [ABC permease protein sufD] |
| *PG0263* | tyrosyl-tRNA synthetase |
| *PG0264* | glycosyl transferase, group 2 family protein |
| *PG0265* | hypothetical protein |
| *PG0267* | arginyl-tRNA synthetase |
| *PG0268* | tRNA (5-methylaminomethyl-2-thiouridylate)-methyltransferase |
| *PG0269* | exodeoxyribonuclease III [only in *Haemophilus influenzae*] |
| *PG0270* | redox-sensitive transcriptional activator OxyR |
| *PG0271* | single-stranded binding protein |
| *PG0272* | CBS domain protein |
| *PG0273* | 4-phosphopantetheinyl transferase family protein |
| *PG0274* | hypothetical protein |
| *PG0275* | thioredoxin family protein |
| *PG0276* | conserved hypothetical protein |
| *PG0278* | hypothetical protein [membrane protease] |
| *PG0279* | NADP-dependent malic enzyme [maeB] |
| *PG0281* | ABC transporter, permease protein, putative |
| *PG0282* | ABC transporter, ATP-binding protein |
| *PG0283* | efflux transporter, MFP component, RND family |
| *PG0285* | hypothetical protein [outer membrane efflux protein] |
| *PG0286* | hypothetical protein |
| *PG0287* | hypothetical protein [porP] |
| *PG0288* | lipoprotein, putative [porK/gdlK] |
| *PG0289* | hypothetical protein [porL/gdlL] |
| *PG0290* | hypothetical protein [gdlM] |
| *PG0291* | hypothetical protein [gdlN] |
| *PG0293* | secretion activator protein, putative |
| *PG0294* | glycosyl transferase, group 2 family protein |
| *PG0295* | DNA processing protein DprA, putative [only in *Helicobacter pylori*] |
| *PG0296* | phosphoribosylformylglycinamidine synthase [purL] |
| *PG0302* | hypothetical protein |
| *PG0303* | electron transport complex, RnfABCDGE type, B subunit |
| *PG0304* | electron transport complex, RnfABCDGE type, C subunit |
| *PG0305* | electron transport complex, RnfABCDGE type, D subunit |
| *PG0306* | electron transport complex, RnfABCDGE type, G subunit |
| *PG0307* | electron transport complex, RnfABCDGE type, E subunit |
| *PG0308* | electron transport complex, RnfABCDGE type, A subunit |
| *PG0309* | thiamine biosynthesis lipoprotein ApbE |
| *PG0310* | nitroreductase family protein |
| *PG0311* | glycosyl transferase, group 2 family protein |
| *PG0312* | hypothetical protein |
| *PG0313* | hypothetical protein [only in *Salmonella*] |
| *PG0314* | ribosomal protein L21 |
| *PG0315* | ribosomal protein L27 |
| *PG0316* | seryl-tRNA synthetase |
| *PG0319* | hypothetical protein [lipoprotein] |
| *PG0320* | hypothetical protein [lipoprotein] |
| *PG0321* | LAO-AO transport system ATPase |
| *PG0322* | serine-threonine transporter |
| *PG0323* | conserved hypothetical protein |
| *PG0324* | histidine ammonia-lyase [hutH] |
| *PG0325* | conserved hypothetical protein [methenyltetrahydrofolate cyclohydrolase] |
| *PG0326* | hypothetical protein [phosphate-selective porin O and P] |
| *PG0327* | hypothetical protein [transporter gate domain protein] |
| *PG0328* | imidazolonepropionase |
| *PG0329* | formiminotransferase-cyclodeaminase-related protein |
| *PG0330* | DNA-binding protein, histone-like family |
| *PG0332* | transcription termination factor Rho |
| *PG0333* | membrane protein, putative |
| *PG0334* | glycosyl transferase, group 2 family protein |
| *PG0335* | tRNA delta(2)-isopentenylpyrophosphate transferase |
| *PG0336* | hypothetical protein |
| *PG0337* | hypothetical protein |
| *PG0338* | hypothetical protein |
| *PG0339* | hypothetical protein |
| *PG0343* | methionine gamma-lyase |
| *PG0344* | purple acid phosphatase |
| *PG0345* | hypothetical protein |
| *PG0346* | GTP-binding protein |
| *PG0347* | UDP-glucose 4-epimerase |
| *PG0348* | ATP-dependent DNA helicase RecG |
| *PG0350* | internalin-related protein |
| *PG0351* | hypothetical protein |
| *PG0352* | sialidase, putative |
| *PG0355* | hypothetical protein |
| *PG0356* | conserved hypothetical protein [lipoprotein] |
| *PG0357* | aspartate carbamoyltransferase, catalytic subunit |
| *PG0358* | aspartate carbamoyltransferase, regulatory subunit [only in *Vibrio cholerae*] |
| *PG0359* | flavin reductase domain protein |
| *PG0360* | lemA protein |
| *PG0361* | conserved domain protein |
| *PG0362* | hypothetical protein |
| *PG0363* | conserved domain protein |
| *PG0364* | conserved hypothetical protein |
| *PG0365* | 3-5 exonuclease domain protein |
| *PG0366* | hypothetical protein |
| *PG0368* | DNA topoisomerase IV, B subunit, putative |
| *PG0369* | phosphopantetheine adenylyltransferase |
| *PG0371* | hypothetical protein |
| *PG0373* | hypothetical protein |
| *PG0374* | hypothetical protein |
| *PG0376* | ribosomal protein S9 |
| *PG0377* | ribosomal protein S2 |
| *PG0378* | translation elongation factor Ts |
| *PG0380* | excinuclease ABC, B subunit |
| *PG0381* | sodium-hydrogen antiporter [potasium/proton antiporter] |
| *PG0383* | membrane-associated zinc metalloprotease, putative |
| *PG0384* | MutS2 family protein |
| *PG0385* | ribosomal protein S21 |
| *PG0386* | site-specific recombinase, phage integrase family-ribosomal subunit interface protein |
| *PG0387* | translation elongation factor Tu |
| *PG0389* | transcription antitermination protein NusG |
| *PG0390* | ribosomal protein L11 |
| *PG0391* | ribosomal protein L1 |
| *PG0392* | ribosomal protein L10 |
| *PG0393* | ribosomal protein L7-L12 |
| *PG0394* | DNA-directed RNA polymerase, beta subunit |
| *PG0395* | DNA-directed RNA polymerase, beta subunit |
| *PG0396* | transcriptional regulator, Crp-Fnr family |
| *PG0397* | hypothetical protein |
| *PG0398* | recF protein |
| *PG0399* | lipoprotein, putative [gliding motility protein gdlH] |
| *PG0400* | conserved hypothetical protein |
| *PG0401* | KH-HDIG domain protein |
| *PG0403* | hypothetical protein |
| *PG0404* | hypothetical protein |
| *PG0408* | hypothetical protein |
| *PG0409* | hypothetical protein |
| *PG0412* | DNA mismatch repair protein MutL |
| *PG0413* | hypothetical protein [membrane protein] |
| *PG0414* | hypothetical protein [ostA-like protein] |
| *PG0415* | peptidyl-prolyl cis-trans isomerase, PPIC-type |
| *PG0416* | ATP-dependent DNA helicase RecQ |
| *PG0417* | ATP-dependent Clp protease, ATP-binding subunit ClpX |
| *PG0418* | ATP-dependent Clp protease, proteolytic subunit |
| *PG0419* | hypothetical protein |
| *PG0422* | hypothetical protein |
| *PG0423* | hypothetical protein |
| *PG0424* | hypothetical protein [integral membrane protein] |
| *PG0425* | esterase, putative |
| *PG0428* | hypothetical protein |
| *PG0429* | pyruvate synthase [only in *Helicobacter pylori*] |
| *PG0430* | oxidoreductase, putative [only in *Helicobacter pylori*] |
| *PG0431* | hypothetical protein |
| *PG0432* | NOL1-NOP2-sun family protein |
| *PG0433* | tetrapyrrole methylase family protein |
| *PG0434* | hypothetical protein |
| *PG0435* | capsular polysaccharide biosythesis protein, putative [only in *Streptococcus pneumoniae*] |
| *PG0437* | polysaccharide export protein, BexD-CtrA-VexA family |
| *PG0438* | hypothetical protein |
| *PG0441* | hypothetical protein |
| *PG0443* | hemagglutinin-related protein |
| *PG0445* | peptidase T [only in *Haemophilus influenzae*] |
| *PG0446* | thiF protein |
| *PG0447* | conserved hypothetical protein [pantothenate kinase] |
| *PG0448* | hypothetical protein [outer membrane protein] |
| *PG0449* | TPR domain protein |
| *PG0450* | hypothetical protein [lipoprotein] |
| *PG0451* | CBS domain protein |
| *PG0452* | conserved hypothetical protein [peptidyl-prolyl cis-trans isomerase] |
| *PG0453* | conserved domain protein [hemolysin] |
| *PG0462* | transporter, putative |
| *PG0463* | folylpolyglutamate synthase |
| *PG0464* | adenylosuccinate synthetase |
| *PG0465* | ferric uptake transcriptional regulator |
| *PG0466* | hypothetical protein |
| *PG0468* | mannose-6-phosphate isomerase, class I |
| *PG0469* | hypothetical protein |
| *PG0470* | hypothetical protein |
| *PG0471* | hypothetical protein |
| *PG0472* | iron-sulfur cluster binding protein, putative |
| *PG0474* | low-specificity L-threonine aldolase |
| *PG0475* | oxygen-independent coproporphyrinogen III oxidase, putative |
| *PG0476* | yngK protein |
| *PG0477* | pantoate--beta-alanine ligase |
| *PG0479* | hypothetical protein |
| *PG0481* | 2-amino-3-ketobutyrate CoA ligase [5-aminolevulinate synthase] |
| *PG0482* | hypothetical protein |
| *PG0483* | kinase, putative |
| *PG0484* | hypothetical protein |
| *PG0485* | preprotein translocase, YajC subunit |
| *PG0486* | methylated-DNA--protein-cysteine S-methyltransferase |
| *PG0488* | Holliday junction DNA helicase RuvB |
| *PG0489* | polysaccharide biosynthesis-related protein |
| *PG0490* | membrane protein, putative |
| *PG0491* | conserved hypothetical protein [dipeptidyl peptidase 7] |
| *PG0494* | hypothetical protein |
| *PG0495* | hypothetical protein |
| *PG0496* | hypothetical protein |
| *PG0497* | 5-methylthioadenosine-S-adenosylhomocysteine nucleosidase |
| *PG0500* | queuine tRNA-ribosyltransferase |
| *PG0501* | conserved hypothetical protein |
| *PG0502* | SsrA-binding protein |
| *PG0503* | dipeptidyl aminopeptidase IV |
| *PG0504* | lipoate synthase [lipA] |
| *PG0505* | hypothetical protein |
| *PG0506* | arginine-specific cysteine proteinase |
| *PG0508* | HAD-superfamily subfamily IB hydrolase, TIGR01490 |
| *PG0509* | prenyltransferase, UbiA family [only in *Mycobacterium tuberculosis*] |
| *PG0510* | conserved hypothetical protein |
| *PG0511* | spore maturation protein A-spore maturation protein B |
| *PG0512* | guanylate kinase |
| *PG0513* | conserved hypothetical protein TIGR00255 |
| *PG0514* | preprotein translocase, SecA subunit |
| *PG0515* | conserved hypothetical protein [alkaline phosphatase] |
| *PG0516* | conserved hypothetical protein |
| *PG0517* | hypothetical protein |
| *PG0518* | abortive infection protein family |
| *PG0519* | hypothetical protein |
| *PG0520* | chaperonin, 60 kDa |
| *PG0521* | chaperonin, 10 kDa |
| *PG0522* | tRNA delta(2)-isopentenylpyrophosphate transferase |
| *PG0523* | inosine-5-monophosphate dehydrogenase |
| *PG0524* | hypothetical protein |
| *PG0525* | CTP synthase |
| *PG0526* | membrane protein, putative [yidC] |
| *PG0528* | amidophosphoribosyltransferase, putative |
| *PG0529* | carbamoyl-phosphate synthase, small subunit |
| *PG0530* | carbamoyl-phosphate synthase, large subunit |
| *PG0531* | glutamine-dependent NAD+ synthetase |
| *PG0532* | conserved domain protein |
| *PG0534* | hypothetical protein [tonB-dependent protein] |
| *PG0535* | conserved hypothetical protein [outer membrane protein chaperone] |
| *PG0536* | hypothetical protein |
| *PG0537* | aminoacyl-histidine dipeptidase [only in *Haemophilus influenzae*] |
| *PG0538* | outer membrane efflux protein |
| *PG0539* | efflux transporter, MFP component, RND family |
| *PG0540* | AcrB-AcrD-AcrF family protein [only in *Salmonella*] |
| *PG0541* | hypothetical protein |
| *PG0547* | conserved hypothetical protein |
| *PG0548* | pyruvate ferredoxin-flavodoxin oxidoreductase family protein |
| *PG0553* | extracellular protease, putative [por secretion system] |
| *PG0554* | hypothetical protein |
| *PG0555* | DNA-binding protein, histone-like family |
| *PG0558* | purine nucleoside phosphorylase I, inosine and guanosine-specific |
| *PG0559* | chlorohydrolase family protein |
| *PG0561* | peptidase, M20-M25-M40 family |
| *PG0562* | potassium uptake protein TrkA, putative [only in *Haemophilus influenzae*] |
| *PG0568* | translation elongation factor P |
| *PG0571* | aspartate-semialdehyde dehydrogenase |
| *PG0572* | hypothetical protein |
| *PG0573* | S-adenosyl-methyltransferase MraW |
| *PG0574* | hypothetical protein |
| *PG0575* | penicillin-binding protein 2, putative |
| *PG0576* | UDP-N-acetylmuramoylalanyl-D-glutamyl-2, 6-diaminopimelate ligase [murE] |
| *PG0577* | phospho-N-acetylmuramoyl-pentapeptide-transferase |
| *PG0578* | UDP-N-acetylmuramoylalanine--D-glutamate ligase |
| *PG0579* | cell division protein FtsW, putative [rodA] |
| *PG0580* | UDP-N-acetylglucosamine--N-acetylmuramyl-(pentapeptide) pyrophosphoryl-undecaprenol N-acetylglucosamine transferase |
| *PG0581* | UDP-N-acetylmuramate--alanine ligase |
| *PG0582* | cell division protein FtsQ, putative |
| *PG0583* | cell division protein FtsA |
| *PG0584* | cell division protein FtsZ |
| *PG0585* | YqeY family protein [only in *Vibrio cholerae*] |
| *PG0587* | yadS protein |
| *PG0588* | 3-methyl-2-oxobutanoate hydroxymethyltransferase |
| *PG0589* | GMP synthase |
| *PG0592* | ribosomal protein L31 |
| *PG0593* | htrA protein |
| *PG0594* | RNA polymerase sigma-70 factor [rpoD] |
| *PG0595* | ribosomal protein S6 |
| *PG0596* | ribosomal protein S18 |
| *PG0597* | ribosomal protein L9 |
| *PG0598* | hypothetical protein [permease] |
| *PG0599* | 3,4-dihydroxy-2-butanone 4-phosphate synthase-GTP cyclohydrolase II |
| *PG0602* | hypothetical protein [por secretion system protein porQ] |
| *PG0605* | hypothetical protein [endonuclease/exonuclease/phosphatase family] |
| *PG0606* | hypothetical protein |
| *PG0607* | hypothetical protein [alkyl hydroperoxide reductase/ Thiol specific antioxidant] |
| *PG0608* | hypothetical protein |
| *PG0610* | hypothetical protein |
| *PG0612* | hypothetical protein |
| *PG0613* | hypothetical protein |
| *PG0615* | GTP-binding protein TypA |
| *PG0616* | thioredoxin, putative |
| *PG0618* | alkyl hydroperoxide reductase, C subunit |
| *PG0619* | alkyl hydroperoxide reductase, F subunit |
| *PG0620* | ATP-dependent protease La |
| *PG0621* | conserved hypothetical protein |
| *PG0622* | hypothetical protein |
| *PG0623* | triosephosphate isomerase |
| *PG0624* | hypothetical protein |
| *PG0625* | GTP cyclohydrolase I |
| *PG0627* | RNA-binding protein |
| *PG0628* | ABC transporter, ATP-binding protein |
| *PG0629* | ATP-NAD kinase |
| *PG0630* | pyridoxal phosphate biosynthetic protein PdxJ |
| *PG0631* | MotA-TolQ-ExbB proton channel family protein [only in *Vibrio cholerae*] |
| *PG0632* | biopolymer transport protein ExbD, putative |
| *PG0633* | hypothetical protein |
| *PG0634* | ThiJ-PfpI family protein [only in *Vibrio cholerae*] |
| *PG0635* | ribosomal protein L11 methyltransferase [only in *Haemophilus influenzae*] |
| *PG0636* | MATE efflux family protein |
| *PG0637* | thiamine monophosphate kinase |
| *PG0638* | tetraacyldisaccharide 4-kinase |
| *PG0639* | signal peptide peptidase SppA, 67K type |
| *PG0644* | TonB-linked receptor Tlr, authentic frameshift |
| *PG0645* | conserved domain protein |
| *PG0646* | iron compound ABC transporter, ATP-binding protein |
| *PG0647* | iron compound ABC transporter, permease protein |
| *PG0648* | iron compound ABC transporter, periplasmic iron compound-binding protein, putative |
| *PG0649* | hypothetical protein |
| *PG0650* | hypothetical protein |
| *PG0651* | HDIG domain protein |
| *PG0652* | conserved hypothetical protein |
| *PG0653* | phosphoserine phosphatase [serB] |
| *PG0654* | hypothetical protein [por secretion system] |
| *PG0656* | ribosomal protein L34 |
| *PG0657* | maf protein [only in *Salmonella*] |
| *PG0658* | phosphatase, YrbI family [only in *Helicobacter pylori*] |
| *PG0659* | conserved hypothetical protein [pyrroline-5-carboxylate reductase] |
| *PG0660* | nitroreductase family protein |
| *PG0661* | hypothetical protein |
| *PG0664* | oxidoreductase, Gfo-Idh-MocA family |
| *PG0665* | beta-galactosidase |
| *PG0666* | mdsC protein, authentic frameshift |
| *PG0668* | TonB-dependent receptor [only in *Acinetobacter baylyi*] |
| *PG0669* | heme-binding protein FetB |
| *PG0670* | lipoprotein, putative |
| *PG0671* | iron compound ABC transporter, permease protein [fepD] |
| *PG0672* | iron compound ABC transporter, ATP-binding protein [fepC] |
| *PG0674* | indolepyruvate ferredoxin oxidoreductase, beta subunit [iorB] |
| *PG0675* | indolepyruvate ferredoxin oxidoreductase, alpha subunit [iorA] |
| *PG0676* | oxidoreductase, short chain dehydrogenase-reductase family |
| *PG0677* | saccharopine dehydrogenase |
| *PG0678* | pyrazinamidase-nicotinamidase, putative |
| *PG0679* | outer membrane efflux protein |
| *PG0680* | efflux transporter, MFP component, RND family |
| *PG0682* | ABC transporter, permease protein, putative |
| *PG0684* | ABC transporter, permease protein, putative |
| *PG0685* | ABC transporter, ATP-binding protein [lolD] |
| *PG0686* | conserved hypothetical protein [PAS/PAC sensor protein] |
| *PG0687* | succinate-semialdehyde dehydrogenase |
| *PG0689* | NAD-dependent 4-hydroxybutyrate dehydrogenase [adhE] |
| *PG0690* | 4-hydroxybutyrate CoA-transferase |
| *PG0691* | NifU-related protein |
| *PG0692* | 4-hydroxybutyryl-CoA dehydratase |
| *PG0694* | immunoreactive 42 kDa antigen PG33 |
| *PG0695* | immunoreactive 43 kDa antigen PG32 |
| *PG0698* | lipoprotein, putative |
| *PG0700* | hypothetical protein |
| *PG0701* | cobinamide kinase-cobinamide phosphate guanylyltransferase |
| *PG0702* | nicotinate-nucleotide--dimethylbenzimidazole phosphoribosyltransferase, putative |
| *PG0703* | cobalamin (5-phosphate) synthase, putative |
| *PG0704* | phosphoglycerate mutase family protein |
| *PG0705* | glutamate racemase [murI] |
| *PG0706* | hypothetical protein [META domain protein] |
| *PG0707* | TonB-dependent receptor, putative [only in *Acinetobacter baylyi*] |
| *PG0708* | peptidyl-prolyl cis-trans isomerase, FKBP-type [only in *Vibrio cholerae*] |
| *PG0709* | peptidyl-prolyl cis-trans isomerase FkpA, FKBP-type [only in *Vibrio cholerae*] |
| *PG0710* | peptidyl-prolyl cis-trans isomerase, FKBP-type [only in *Vibrio cholerae*] |
| *PG0711* | hypothetical protein |
| *PG0712* | hypothetical protein |
| *PG0713* | anthranilate synthase component II [trpG] |
| *PG0714* | copper homeostasis protein CutC [only in *E. coli*] |
| *PG0715* | transporter |
| *PG0720* | DNA-binding response regulator |
| *PG0721* | NLP-P60 family protein [only in *Haemophilus influenzae*] |
| *PG0722* | hypothetical protein |
| *PG0723* | hypothetical protein |
| *PG0724* | prolyl oligopeptidase family protein [tolB] |
| *PG0725* | hydrolase, haloacid dehalogenase-like family |
| *PG0726* | lipoprotein, putative |
| *PG0727* | hypothetical protein |
| *PG0728* | conserved hypothetical protein |
| *PG0729* | D-alanine--D-alanine ligase |
| *PG0730* | ribosomal large subunit pseudouridine synthase D |
| *PG0731* | hypothetical protein [PASTA domain protein] |
| *PG0732* | hypothetical protein |
| *PG0733* | riboflavin synthase, alpha subunit |
| *PG0734* | nitroreductase family protein |
| *PG0735* | aminotransferase, class V |
| *PG0736* | ribonuclease HII [rnhB] |
| *PG0737* | hypothetical protein |
| *PG0738* | cytidine-deoxycytidylate deaminase family protein |
| *PG0739* | metallo-beta-lactamase family protein [ribonuclease Z] |
| *PG0740* | NLP-P60 family protein |
| *PG0744* | RNA methyltransferase, TrmH family |
| *PG0745* | lactoylglutathione lyase, putative |
| *PG0746* | sensor histidine kinase |
| *PG0747* | sigma-54 dependent DNA-binding response regulator [only in *Vibrio cholera*] |
| *PG0749* | hypothetical protein |
| *PG0750* | glycosyl transferase, group 2 family protein |
| *PG0751* | porT protein |
| *PG0752* | uracil phosphoribosyltransferase, putative |
| *PG0753* | Protease [only in *Streptococcus pneumoniae*] |
| *PG0754* | DNA topoisomerase I |
| *PG0756* | conserved hypothetical protein [only in *Mycoplasma genitalium*] |
| *PG0757* | hypothetical protein |
| *PG0758* | peptidyl-dipeptidase Dcp |
| *PG0759* | TPR domain protein |
| *PG0762* | trigger factor, putative |
| *PG0766* | polyribonucleotide nucleotidyltransferase |
| *PG0767* | 4-alpha-glucanotransferase [only in *Haemophilus influenzae*] |
| *PG0768* | conserved hypothetical protein |
| *PG0769* | fibronectin type III domain protein |
| *PG0770* | hypothetical protein |
| *PG0771* | hypothetical protein |
| *PG0774* | hypothetical protein |
| *PG0775* | acyl-CoA dehydrogenase family protein |
| *PG0776* | electron transfer flavoprotein, alpha subunit |
| *PG0777* | electron transfer flavoprotein, beta subunit |
| *PG0778* | conserved hypothetical protein |
| *PG0779* | hypothetical protein [transport energizing protein, ExbD/TolR family] |
| *PG0780* | hypothetical protein [biopolymer transport protein ExbD/TolR] |
| *PG0781* | hypothetical protein |
| *PG0782* | MotA-TolQ-ExbB proton channel family protein |
| *PG0783* | hydrolase, putative |
| *PG0784* | polyprenyl synthetase |
| *PG0787* | hypothetical protein |
| *PG0788* | hypothetical protein [Cna protein B-type domain protein] |
| *PG0789* | conserved hypothetical protein [phosphoenolpyruvate synthase] |
| *PG0790* | GTP-binding protein Obg |
| *PG0791* | adenylate kinase |
| *PG0792* | hypoxanthine phosphoribosyltransferase |
| *PG0793* | fructose-1,6-bisphosphatase |
| *PG0794* | penicillin-binding protein 1A, putative |
| *PG0795* | membrane protein, putative |
| *PG0796* | leucyl-tRNA synthetase |
| *PG0799* | hypothetical protein |
| *PG0800* | conserved hypothetical protein [NAD-utilizing dehydrogenase] |
| *PG0801* | polyA polymerase family protein |
| *PG0802* | alpha keto acid dehydrogenase complex, E3 component, lipoamide dehydrogenase |
| *PG0803* | glucosamine-6-phosphate isomerase |
| *PG0804* | flavodoxin |
| *PG0805* | prolipoprotein diacylglyceryl transferase |
| *PG0806* | oxidoreductase, Gfo-Idh-MocA family |
| *PG0807* | NusB family protein |
| *PG0810* | hypothetical protein |
| *PG0811* | Holliday junction DNA helicase RuvA |
| *PG0812* | ISPg9, transposase, degenerate |
| *PG0813* | ISPg1, transposase, truncation [only in *E. coli*] |
| *PG0876* | thiophene and furan oxidation protein ThdF [tRNA modification GTPase TrmE] |
| *PG0877* | hypothetical protein [gldB] |
| *PG0881* | recA protein |
| *PG0882* | hypothetical protein [vimA] |
| *PG0883* | hypothetical protein [vimE] |
| *PG0884* | hypothetical protein [vimF] |
| *PG0885* | phospho-2-dehydro-3-deoxyheptonate aldolase-chorismate mutase |
| *PG0886* | hypothetical protein [2-amino-4-hydroxy-6-hydroxymethyldihydropteridine diphosphokinases] |
| *PG0888* | hypothetical protein |
| *PG0889* | peptidase, M24 family [pepP] |
| *PG0890* | alkaline phosphatase, putative |
| *PG0893* | prismane protein [Hydroxylamine reductase] |
| *PG0894* | DNA repair protein RadC [only in *Streptococcus pneumoniae*] |
| *PG0896* | beta-galactosidase |
| *PG0897* | alpha-amylase family protein [only in *Vibrio cholerae*] |
| *PG0898* | conserved hypothetical protein [only in *Salmonella*] |
| *PG0899* | cytochrome d ubiquinol oxidase, subunit II |
| *PG0900* | cytochrome d ubiquinol oxidase, subunit I |
| *PG0901* | conserved hypothetical protein |
| *PG0902* | alpha-1,2-mannosidase family protein |
| *PG0903* | conserved hypothetical protein [arginine decarboxylase, pyruvoyl-dependent] |
| *PG0906* | lipoprotein, putative |
| *PG0908* | G-U mismatch-specific DNA glycosylase, putative |
| *PG0909* | conserved hypothetical protein [only in *Haemophilus influenzae*] |
| *PG0910* | FHA domain protein |
| *PG0912* | polysaccharide transport protein, putative |
| *PG0914* | hypothetical protein |
| *PG0915* | conserved hypothetical protein [3-methylpurine-DNA glycosylase] |
| *PG0917* | GtrA family protein |
| *PG0918* | hypothetical protein |
| *PG0919* | dihydroorotase |
| *PG0920* | glycosyl transferase, group 2 family protein |
| *PG0922* | membrane protein, putative |
| *PG0923* | ribosome-binding factor A |
| *PG0924* | 5-nucleotidase, lipoprotein e(P4) family |
| *PG0925* | thymidine kinase |
| *PG0926* | hypothetical protein |
| *PG0927* | conserved hypothetical protein TIGR00150 |
| *PG0928* | response regulator |
| *PG0930* | hypothetical protein |
| *PG0931* | DNA-binding protein, histone-like family, degenerate |
| *PG0932* | DNA polymerase III, delta prime subunit, putative |
| *PG0933* | translation elongation factor G, putative |
| *PG0934* | radical SAM domain protein |
| *PG0935* | 4-diphosphocytidyl-2C-methyl-D-erythritol kinase |
| *PG0936* | xanthine-uracil permease family protein |
| *PG0937* | hypothetical protein |
| *PG0938* | calcium-transporting ATPase |
| *PG0945* | ABC transporter, permease protein, putative |
| *PG0946* | ABC transporter, ATP-binding protein |
| *PG0948* | AMP nucleosidase, putative |
| *PG0949* | conserved hypothetical protein [DNA polymerase III][only in *Bacillus subtilis*] |
| *PG0950* | glycine cleavage system H protein |
| *PG0951* | phosphoribosylaminoimidazole carboxylase, PurE protein |
| *PG0952* | 1-hydroxy-2-methyl-2-(E)-butenyl 4-diphosphate synthase |
| *PG0953* | deoxyuridine 5-triphosphate nucleotidohydrolase |
| *PG0955* | hypothetical protein |
| *PG0956* | peptidase, M23-M37 family, putative |
| *PG0957* | riboflavin biosynthesis protein RibF |
| *PG0958* | YihY family protein [ribonuclease BN] [only in *Haemophilus influenzae*] |
| *PG0959* | ATP-binding protein, Mrp-Nbp35 family |
| *PG0960* | conserved hypothetical protein [tRNA (guanine-N(7)-)-methyltransferase] |
| *PG0961* | hypothetical protein |
| *PG0962* | prolyl-tRNA synthetase |
| *PG0963* | hypothetical protein |
| *PG0964* | CDP-diacylglycerol--serine O-phosphatidyltransferase, putative, authentic point mutation |
| *PG0965* | phosphatidylserine decarboxylase-related protein |
| *PG0969* | S-adenosylmethionine:tRNA ribosyltransferase-isomerase, putative |
| *PG0973* | alpha-1,2-mannosidase family protein |
| *PG0975* | PhoH family protein [only in *Vibrio cholerae*] |
| *PG0976* | phosphoribosylaminoimidazole-succinocarboxamide synthase, putative |
| *PG0977* | ubiquinone-menaquinone biosynthesis methyltransferase UbiE |
| *PG0978* | shikimate 5-dehydrogenase |
| *PG0980* | hypothetical protein [outer membrane protein, OMP85 family] |
| *PG0984* | hypothetical protein |
| *PG0985* | RNA polymerase sigma-70 factor, ECF subfamily |
| *PG0986* | hypothetical protein |
| *PG0987* | hypothetical protein |
| *PG0989* | ribosomal protein L20 |
| *PG0990* | ribosomal protein L35 |
| *PG0991* | translation initiation factor IF-3 |
| *PG0992* | threonyl-tRNA synthetase |
| *PG0995* | hypothetical protein |
| *PG0996* | conserved hypothetical protein TIGR01777 [only in *E. coli*] |
| *PG0997* | transcriptional regulator, putative |
| *PG0999* | hypothetical protein |
| *PG1000* | hypothetical protein [HEAT repeat protein] |
| *PG1001* | conserved hypothetical protein [Lin2532-like protein] |
| *PG1003* | conserved hypothetical protein [nitroreductase] [only in *Mycobacterium tuberculosis*] |
| *PG1004* | prolyl oligopeptidase family protein |
| *PG1005* | lipoprotein, putative |
| *PG1006* | hypothetical protein |
| *PG1007* | transcriptional regulator, GntR family |
| *PG1008* | hypothetical protein |
| *PG1009* | hypothetical protein |
| *PG1012* | tRNA-i(6)A37 modification enzyme MiaB |
| *PG1013* | acetyl-CoA hydrolase-transferase family protein |
| *PG1017* | pyruvate phosphate dikinase |
| *PG1019* | lipoprotein, putative |
| *PG1020* | hypothetical protein |
| *PG1022* | hypothetical protein |
| *PG1023* | para-aminobenzoate synthase, component I, authentic point mutation |
| *PG1024* | hypothetical protein |
| *PG1027* | hypothetical protein |
| *PG1030* | hypothetical protein [por secretion system protein] |
| *PG1033* | conserved hypothetical protein [permease] |
| *PG1034* | ABC transporter, ATP-binding protein |
| *PG1035* | hypothetical protein |
| *PG1036* | excinuclease ABC, A subunit [uvrA] |
| *PG1037* | hypothetical protein |
| *PG1038* | ATP-dependent DNA helicase UvrD-PcrA-Rep Family |
| *PG1039* | integral membrane protein |
| *PG1041* | K+-dependent Na+-Ca+ exchanger related-protein |
| *PG1042* | glycogen synthase, putative |
| *PG1043* | ferrous iron transport protein B [only in *Caulobacter crescentus*] |
| *PG1044* | iron dependent repressor, putative |
| *PG1048* | N-acetylmuramoyl-L-alanine amidase, family 3 |
| *PG1049* | conserved hypothetical protein |
| *PG1050* | hypothetical protein [lipoprotein] |
| *PG1051* | hypothetical protein [O-antigen ligase, waaL] |
| *PG1052* | transcriptional regulator, putative |
| *PG1053* | transcriptional regulator, putative |
| *PG1056* | conserved hypothetical protein [6-pyruvoyl tetrahydrobiopterin synthase] |
| *PG1057* | conserved hypothetical protein [nrdG] [only in *Haemophilus influenzae*] |
| *PG1058* | OmpA family protein [only in *Vibrio cholerae*] |
| *PG1060* | carboxyl-terminal protease [only in *Salmonella*] |
| *PG1064* | dihydroorotate dehydrogenase, putative |
| *PG1065* | dihydroorotate dehydrogenase [only in *Acinetobacter baylyi*] |
| *PG1066* | butyrate-acetoacetate CoA-transferase, subunit A [only in *Haemophilus influenzae* ] |
| *PG1067* | conserved hypothetical protein [beta-alanyl-CoA:ammonia lyase] |
| *PG1068* | conserved hypothetical protein [transposase] |
| *PG1069* | alcohol dehydrogenase, zinc-containing, putative |
| *PG1070* | L-lysine 2,3-aminomutase [only in *Haemophilus influenzae*] |
| *PG1071* | conserved hypothetical protein |
| *PG1072* | MutS family protein |
| *PG1073* | D-lysine 5,6-aminomutase, alpha subunit |
| *PG1074* | D-lysine 5,6-aminomutase, beta subunit |
| *PG1075* | coenzyme A transferase, beta subunit [only in *Haemophilus influenzae*] |
| *PG1076* | acyl-CoA dehydrogenase, short-chain specific |
| *PG1077* | electron transfer flavoprotein, beta subunit |
| *PG1078* | electron transfer flavoprotein, alpha subunit |
| *PG1079* | enoyl-CoA hydratase-isomerase family protein |
| *PG1080* | 3-hydroxyacyl-CoA dehydrogenase family protein [only in *Mycobacterium tuberculosis*] |
| *PG1081* | acetate kinase |
| *PG1082* | phosphotransacetylase |
| *PG1083* | hypothetical protein |
| *PG1084* | thioredoxin family protein [only in *Bacillus subtilis*] |
| *PG1085* | hypothetical protein |
| *PG1087* | radical SAM protein, TIGR01212 family |
| *PG1088* | acetyltransferase, GNAT family |
| *PG1089* | DNA-binding response regulator RprY |
| *PG1091* | DHH subfamily 1 protein |
| *PG1093* | hypothetical protein |
| *PG1094* | phosphomannomutase |
| *PG1095* | RNA methyltransferase, TrmA family [only in *S. aureus*] |
| *PG1096* | hypothetical protein [GSCFA family protein] |
| *PG1097* | Mur ligase domain protein-alanine racemase |
| *PG1098* | hypothetical protein [SAM-dependent methyltransferase] |
| *PG1099* | glucokinase regulator-related protein [N-acetylmuramic acid 6-phosphate etherase] |
| *PG1100* | hypothetical protein [BadF/BadG/BcrA/BcrD ATPase domain protein] |
| *PG1101* | sodium:solute symporter family protein [only in *Salmonella*] |
| *PG1103* | ATPase, AAA family |
| *PG1104* | conserved hypothetical protein [tRNA-methyltransferase] [only in *Haemophilus influenzae*] |
| *PG1105* | RNA polymerase sigma-54 factor |
| *PG1106* | UDP-N-acetylmuramoylalanyl-D-glutamyl-2,6-diaminopimelate--D-alanyl-D-alanyl ligase |
| *PG1114* | aspartate-1-decarboxylase |
| *PG1116* | methylenetetrahydrofolate dehydrogenase-methenyltetrahydrofolate cyclohydrolase |
| *PG1117* | MATE efflux family protein [only in *Haemophilus influenzae*] |
| *PG1118* | clpB protein |
| *PG1121* | asparaginyl-tRNA synthetase |
| *PG1122* | ribosomal large subunit pseudouridine synthase B |
| *PG1123* | adenylosuccinate lyase |
| *PG1124* | ATP:cob(I)alamin adenosyltransferase, putative |
| *PG1125* | hypothetical protein |
| *PG1126* | uracil permease [only in *S. aureus*] |
| *PG1127* | transcriptional regulator, AsnC Family [only in *Pseudomonas aeruginosa*] |
| *PG1128* | exodeoxyribonuclease VII, large subunit |
| *PG1129* | ribonucleotide reductase |
| *PG1132* | valyl-tRNA synthetase |
| *PG1133* | hypothetical protein |
| *PG1134* | thioredoxin reductase |
| *PG1135* | bacterial sugar transferase [only in *Streptococcus pneumoniae*] |
| *PG1136* | conserved hypothetical protein [asparagine synthase family] |
| *PG1137* | porS protein |
| *PG1138* | pigmentation and extracellular proteinase regulator |
| *PG1139* | hypothetical protein |
| *PG1140* | glycosyl transferase, group 2 family protein [wbbL] [only in *Mycobacterium tuberculosis*] |
| *PG1142* | exopolysaccharide synthesis-related protein |
| *PG1144* | peptide chain release factor 2, programmed frameshift |
| *PG1151* | alcohol dehydrogenase, iron-containing [only in *Streptococcus pneumoniae*] |
| *PG1152* | hypothetical protein |
| *PG1153* | hypothetical protein |
| *PG1155* | ADP-heptose--LPS heptosyltransferase, putative |
| *PG1156* | S4 domain protein |
| *PG1159* | cobalamin biosynthesis protein CbiB |
| *PG1160* | L-threonine-O-3-phosphate decarboxylase, putative |
| *PG1161* | cobyric acid synthase CobQ, authentic frameshift |
| *PG1162* | ATP:cob(I)alamin adenosyltransferase, putative |
| *PG1163* | cobyrinic acid a,c-diamide synthase |
| *PG1169* | hypothetical protein |
| *PG1171* | oxidoreductase, putative |
| *PG1172* | iron-sulfur cluster binding protein, putative |
| *PG1173* | YkgG family protein |
| *PG1174* | thioesterase family protein |
| *PG1175* | ABC transporter, ATP-binding protein, putative |
| *PG1176* | ABC transporter, ATP-binding protein, putative |
| *PG1178* | hypothetical protein |
| *PG1179* | conserved hypothetical protein [sigma E regulatory protein, MucB/RseB] |
| *PG1180* | membrane protein, putative |
| *PG1181* | transcriptional regulator, tetR family |
| *PG1184* | alginate O-acetyltransferase, putative [only in *S. aureus*] |
| *PG1185* | hypothetical protein [lipolytic protein G-D-S-L family] |
| *PG1186* | hypothetical protein [periplasmic protein] |
| *PG1189* | hypothetical protein |
| *PG1190* | glycerate dehydrogenase |
| *PG1195* | 8-amino-7-oxononanoate synthase |
| *PG1196* | hypothetical protein |
| *PG1198* | hypothetical protein |
| *PG1208* | dnaK protein |
| *PG1209* | hypothetical protein |
| *PG1210* | peptidase, M24 family [only in *Mycoplasma*] |
| *PG1211* | hexapeptide transferase family protein [only in *E. coli*] |
| *PG1212* | TPR domain protein |
| *PG1213* | ribonuclease H |
| *PG1214* | hypothetical protein |
| *PG1216* | hypothetical protein |
| *PG1217* | hypothetical protein [RNA polyermase Rpb6] |
| *PG1218* | hypothetical protein [membrane protein] |
| *PG1219* | hypothetical protein [transcriptional acessory protein] |
| *PG1220* | erythronate-4-phosphate dehydrogenase, putative |
| *PG1221* | oxidoreductase, short chain dehydrogenase-reductase family |
| *PG1223* | hypothetical protein |
| *PG1224* | ABC transporter, periplasmic substrate-binding protein, putative, degenerate |
| *PG1225* | ABC transporter, ATP-binding protein |
| *PG1226* | peptidyl-prolyl cis-trans isomerase, cyclophilin-type |
| *PG1229* | hypothetical protein |
| *PG1230* | hypothetical protein |
| *PG1232* | glutamate dehydrogenase, NAD-specific |
| *PG1233* | hypothetical protein |
| *PG1235* | epimerase-reductase, putative |
| *PG1236* | hypothetical protein [hemerythrin HHE cation binding domain protein] |
| *PG1237* | transcriptional regulator, LuxR family [only in *Haemophilus influenzae*] |
| *PG1238* | ribosomal large subunit pseudouridine synthase family protein |
| *PG1239* | 3-oxoacyl-(acyl-carrier-protein) reductase |
| *PG1241* | GTP-binding protein Lepa |
| *PG1242* | replicative DNA helicase |
| *PG1246* | alanyl-tRNA synthetase |
| *PG1247* | 3-dehydroquinate synthase |
| *PG1248* | conserved hypothetical protein [DNA alkylation repair enzyme] |
| *PG1249* | 1-acyl-sn-glycerol-3-phosphate acetyltransferase, putative |
| *PG1251* | hypothetical protein [traJ] |
| *PG1252* | membrane protein, putative |
| *PG1253* | DNA ligase, NAD-dependent |
| *PG1254* | acetyltransferase, GNAT family |
| *PG1255* | recombination protein RecR |
| *PG1256* | ribonuclease, Rne-Rng family |
| *PG1258* | DNA-binding protein HU |
| *PG1259* | anaerobic ribonucleoside-triphosphate reductase activating protein |
| *PG1260* | anaerobic ribonucleoside-triphosphate reductase, putative |
| *PG1268* | hypothetical protein |
| *PG1269* | delta-1-pyrroline-5-carboxylate dehydrogenase |
| *PG1270* | conserved hypothetical protein [amidinotransferase] |
| *PG1271* | acetylornithine aminotransferase, putative |
| *PG1273* | hypothetical protein |
| *PG1277* | UDP-glucose-6 dehydrogenase, putative |
| *PG1278* | phosphoserine aminotransferase |
| *PG1279* | D-isomer specific 2-hydroxyacid dehydrogenase family protein |
| *PG1280* | conserved hypothetical protein |
| *PG1281* | hypothetical protein |
| *PG1282* | conserved hypothetical protein [adenylate cyclase] |
| *PG1283* | conserved hypothetical protein [dipeptidyl peptidase 11] |
| *PG1285* | glucosamine-6-phosphate isomerase, putative [only in *Haemophilus influenzae*] |
| *PG1286* | ferritin |
| *PG1288* | GDP-mannose 4,6-dehydratase |
| *PG1289* | GDP-fucose synthetase |
| *PG1290* | branched-chain amino acid aminotransferase |
| *PG1291* | hypothetical protein |
| *PG1294* | ferrous iron transport protein B [only in *Caulobacter crescentus*] |
| *PG1296* | hypothetical protein |
| *PG1297* | ribosomal protein S1 |
| *PG1301* | hypothetical protein |
| *PG1302* | hypothetical protein |
| *PG1303* | helicase, putative |
| *PG1304* | hypothetical protein |
| *PG1305* | glycine cleavage system P protein [only in *Mycobacterium tuberculosis*] |
| *PG1306* | metallo-beta-lactamase family protein [only in *Mycobacterium tuberculosis*] |
| *PG1307* | glucose-inhibited division protein B [only in *S. aureus*] |
| *PG1308* | hypothetical protein |
| *PG1310* | exsB protein |
| *PG1311* | conserved hypothetical protein [phosphodiesterase family protein] |
| *PG1312* | capA protein, putative |
| *PG1313* | dipeptidase-related protein |
| *PG1314* | chorismate synthase |
| *PG1315* | peptidyl-prolyl cis-trans isomerase SlyD, FKBP-type |
| *PG1316* | hypothetical protein |
| *PG1317* | hypothetical protein |
| *PG1318* | RNA polymerase sigma-70 factor, ECF subfamily |
| *PG1321* | formate--tetrahydrofolate ligase |
| *PG1323* | PhoH family protein [only in *Vibrio cholerae*] |
| *PG1324* | crossover junction endodeoxyribonuclease RuvC [only in *E. coli*] |
| *PG1325* | hypothetical protein |
| *PG1327* | aminotransferase, putative |
| *PG1328* | CoA ligase family protein |
| *PG1329* | ISPg5, transposase Orf2, degenerate |
| *PG1330* | large conductance mechanosensitive channel protein [only in *E. coli*] |
| *PG1331* | NAD(P) transhydrogenase, alpha subunit, authentic frameshift |
| *PG1333* | hypothetical protein |
| *PG1334* | band 7-Mec-2 family protein [only in *Salmonella*] |
| *PG1337* | umuD protein |
| *PG1338* | umuC protein |
| *PG1340* | L-lactate permease [only in *S. aureus*] |
| *PG1341* | hypothetical protein |
| *PG1342* | UDP-N-acetylenolpyruvoylglucosamine reductase |
| *PG1343* | lipoate-protein ligase B |
| *PG1345* | glycosyl transferase, group 1 family protein |
| *PG1346* | glycosyl transferase, group 1 family protein |
| *PG1347* | conserved hypothetical protein [7-cyano-7-deazaguanine reductase] |
| *PG1348* | conserved hypothetical protein TIGR00147 [diacylglycerol kinase] |
| *PG1351* | hypothetical protein |
| *PG1352* | hypothetical protein [polyketide cyclase] |
| *PG1353* | orotate phosphoribosyltransferase |
| *PG1354* | hydrolase, carbon-nitrogen family |
| *PG1355* | acyltransferase, putative |
| *PG1356* | hypothetical protein |
| *PG1358* | acetyltransferase, GNAT family |
| *PG1359* | hypothetical protein |
| *PG1360* | phosphoribosylamine--glycine ligase |
| *PG1361* | dipeptidyl aminopeptidase IV, putative |
| *PG1362* | conserved hypothetical protein [N-6 adenine-specific DNA methylase ] [only in *S. aureus*] |
| *PG1363* | hypothetical protein [traJ] |
| *PG1364* | 1-deoxy-D-xylulose 5-phosphate reductoisomerase |
| *PG1365* | 16S rRNA processing protein RimM, putative |
| *PG1366* | UDP-N-acetylglucosamine 1-carboxyvinyltransferase |
| *PG1367* | hypothetical protein |
| *PG1368* | glucose-6-phosphate isomerase |
| *PG1369* | glycerol-3-phosphate dehydrogenase (NAD(P)+) |
| *PG1370* | lysyl-tRNA synthetase |
| *PG1371* | phosphorylase family protein [only in *Vibrio cholerae*] |
| *PG1372* | hypothetical protein [anaphase-promoting complex, cyclosome, subunit 3] |
| *PG1374* | immunoreactive 47 kDa antigen PG97 |
| *PG1375* | hypothetical protein |
| *PG1378* | A-G-specific adenine glycosylase |
| *PG1379* | ABC transporter, periplasmic substrate-binding protein, putative |
| *PG1380* | ABC transporter, ATP-binding protein |
| *PG1381* | ABC transporter, permease protein |
| *PG1382* | hypothetical protein [only in *Haemophilus influenzae*] |
| *PG1383* | amino acid exporter, putative |
| *PG1385* | TPR domain protein |
| *PG1386* | DNA gyrase, A subunit |
| *PG1387* | hypothetical protein |
| *PG1388* | hypothetical protein |
| *PG1389* | DNA-binding protein, histone-like family |
| *PG1391* | hypothetical protein |
| *PG1392* | rod shape-determining protein RodA, putative |
| *PG1393* | penicillin-binding protein 2, putative |
| *PG1394* | hypothetical protein |
| *PG1395* | cell shape-determining protein MreC, putative |
| *PG1396* | cell shape-determining protein MreB |
| *PG1397* | phosphoribosylaminoimidazolecarboxamide formyltransferase-IMP cyclohydrolase |
| *PG1401* | beta-eliminating lyase [tryptophanase] |
| *PG1402* | AP endonuclease domain protein |
| *PG1403* | rhomboid family protein |
| *PG1404* | rhomboid family protein |
| *PG1405* | hypothetical protein [organic solvent tolerance protein ostA] |
| *PG1406* | ISPg2, transposase, truncation |
| *PG1407* | nitroimidazole resistance protein, putative |
| *PG1408* | heavy metal efflux pump, CzcD family |
| *PG1409* | hypothetical protein |
| *PG1410* | hypothetical protein |
| *PG1411* | potassium uptake protein TrkA, putative [only in *Haemophilus influenzae*] |
| *PG1414* | hypothetical protein [TonB-dependent receptor protein] |
| *PG1416* | enoyl-(acyl-carrier-protein) reductase II [only in *Streptococcus*] |
| *PG1417* | fumarate hydratase class I, anaerobic |
| *PG1418* | DNA polymerase III, gamma and tau subunits |
| *PG1421* | ferredoxin, 4Fe-4S |
| *PG1422* | D-alanyl-D-alanine carboxypeptidase [only in *Haemophilus influenzae*] |
| *PG1423* | hypothetical protein |
| *PG1424* | peptidylarginine deiminase |
| *PG1427* | thiol protease-hemagglutinin PrtT precursor, putative |
| *PG1428* | 6,7-dimethyl-8-ribityllumazine synthase |
| *PG1429* | hypothetical protein |
| *PG1430* | TPR domain protein |
| *PG1431* | DNA-binding response regulator, LuxR family |
| *PG1432* | sensor histidine kinase |
| *PG1433* | hydrolase |
| *PG1434* | 4-diphosphocytidyl-2C-methyl-D-erythritol synthase |
| *PG1536* | cell division protein FtsX, putative |
| *PG1537* | conserved hypothetical protein [membrane protein] |
| *PG1538* | undecaprenol kinase, putative |
| *PG1539* | tRNA pseudouridine synthase B [only in *Haemophilus influenzae*] |
| *PG1540* | S-adenosylmethionine:tRNA ribosyltransferase-isomerase |
| *PG1541* | 2-amino-4-hydroxy-6-hydroxymethyldihydropteridine pyrophosphokinase |
| *PG1543* | thioesterase family protein |
| *PG1544* | yaaA protein [only in *Haemophilus influenzae*] |
| *PG1545* | superoxide dismutase, Fe-Mn |
| *PG1547* | hypothetical protein |
| *PG1548* | thiol protease-hemagglutinin PrtT precursor, authentic frameshift |
| *PG1549* | hypothetical protein |
| *PG1551* | hmuY protein |
| *PG1552* | TonB-dependent receptor HmuR |
| *PG1554* | hypothetical protein |
| *PG1555* | conserved domain protein [only in *Pseudomonas aeruginosa*] |
| *PG1556* | conserved hypothetical protein |
| *PG1559* | glycine cleavage system T protein [only in *Mycobacterium tuberculosis*] |
| *PG1560* | dTDP-glucose 4,6-dehydratase |
| *PG1561* | dTDP-4-dehydrorhamnose reductase |
| *PG1562* | dTDP-4-dehydrorhamnose 3,5-epimerase |
| *PG1563* | glucose-1-phosphate thymidylyltransferase |
| *PG1564* | membrane protein, putative [sulfatase] |
| *PG1565* | 3-deoxy-D-manno-octulosonic-acid transferase, putative |
| *PG1566* | glutamyl-tRNA synthetase |
| *PG1570* | rhodanese-like domain protein |
| *PG1571* | metallo-beta-lactamase superfamily protein [only in *Mycobacterium tuberculosis*] |
| *PG1572* | membrane protein, putative |
| *PG1573* | transcriptional regulator, Crp family |
| *PG1576* | L-aspartate oxidase |
| *PG1577* | nicotinate-nucleotide pyrophosphorylase |
| *PG1578* | quinolinate synthetase complex, subunit A |
| *PG1579* | ATPase, MoxR family [only in *Mycobacterium tuberculosis*] |
| *PG1580* | conserved hypothetical protein [only in *Mycobacterium tuberculosis*] |
| *PG1581* | hypothetical protein |
| *PG1582* | batA protein |
| *PG1583* | batB protein |
| *PG1584* | batC protein |
| *PG1585* | batD protein |
| *PG1586* | batE protein |
| *PG1587* | PAP2 superfamily protein |
| *PG1588* | conserved hypothetical protein |
| *PG1589* | dihydropteroate synthase |
| *PG1592* | HDIG domain protein |
| *PG1593* | shikimate kinase |
| *PG1594* | ComEC-Rec2-related protein |
| *PG1595* | ribulose-phosphate 3-epimerase |
| *PG1596* | isoleucyl-tRNA synthetase, putative |
| *PG1597* | DnaK suppressor protein, putative |
| *PG1598* | lipoprotein signal peptidase, putative |
| *PG1599* | hypothetical protein |
| *PG1600* | membrane protein, putative [acyltransferase] |
| *PG1601* | biotin--acetyl-CoA-carboxylase ligase |
| *PG1602* | conserved hypothetical protein [mmcQ] |
| *PG1603* | HAM1 protein [deoxyribonucleoside-triphosphatase] |
| *PG1604* | immunoreactive 84 kDa antigen PG93 |
| *PG1605* | aminopeptidase C |
| *PG1608* | methylmalonyl-CoA decarboxylase, beta subunit |
| *PG1609* | methylmalonyl-CoA decarboxylase, gamma subunit |
| *PG1610* | hypothetical protein |
| *PG1611* | hypothetical protein |
| *PG1612* | methylmalonyl-CoA decarboxylase, alpha subunit |
| *PG1613* | glyoxalase family protein |
| *PG1614* | fumarate reductase, iron-sulfur protein |
| *PG1615* | fumarate reductase, flavoprotein subunit |
| *PG1616* | conserved hypothetical protein [cytochrome B subunit] |
| *PG1618* | conserved hypothetical protein [dithiobiotin synthetase] [only in *Haemophilus influenzae*] |
| *PG1619* | biotin synthesis protein BioC, putative [only in *Acinetobacter baylyi*] |
| *PG1620* | carboxyl-terminal protease-related protein |
| *PG1622* | DNA topoisomerase IV, A subunit, putative |
| *PG1625* | hypothetical protein |
| *PG1626* | hypothetical protein [outer membrane transport protein] |
| *PG1632* | aldose 1-epimerase |
| *PG1633* | galactokinase |
| *PG1634* | hypothetical protein |
| *PG1635* | hypothetical protein [outer membrane lipoprotein carrier protein LolA] |
| *PG1636* | FtsK-SpoIIIE family protein |
| *PG1638* | thioredoxin family protein |
| *PG1639* | hypothetical protein |
| *PG1640* | DNA-damage-inducible protein F |
| *PG1641* | phosphotyrosine protein phosphatase [only in *Pseudomonas aeruginosa*] |
| *PG1647* | cardiolipin synthetase |
| *PG1648* | RelA-SpoT family protein |
| *PG1651* | TPR domain protein |
| *PG1652* | hypothetical protein [TonB-dependent receptor] |
| *PG1654* | D-alanyl-D-alanine dipeptidase |
| *PG1656* | methylmalonyl-CoA mutase, small subunit |
| *PG1657* | methylmalonyl-CoA mutase, large subunit |
| *PG1661* | hypothetical protein |
| *PG1662* | hypothetical protein |
| *PG1663* | ABC transporter, ATP-binding protein |
| *PG1664* | ABC transporter, permease protein, putative |
| *PG1665* | ABC transporter, permease protein, putative |
| *PG1666* | efflux transporter, MFP component, RND family |
| *PG1667* | outer membrane efflux protein |
| *PG1674* | hemagglutinin protein HagB, degenerate |
| *PG1675* | hypothetical protein |
| *PG1676* | phosphoenolpyruvate carboxykinase (ATP) |
| *PG1677* | phosphoglycerate kinase |
| *PG1678* | hypothetical protein |
| *PG1679* | hypothetical protein [membrane protein] |
| *PG1680* | ABC transporter, ATP-binding protein, authentic frameshift |
| *PG1681* | glycogen debranching enzyme, archaeal type, putative |
| *PG1682* | glycosyl transferase, group 1 family protein |
| *PG1683* | conserved hypothetical protein [glycosyl hydrolase] |
| *PG1684* | hypothetical protein |
| *PG1687* | HIT family protein [only in *Mycoplasma genitalium*] |
| *PG1688* | transcription elongation factor GreA |
| *PG1690* | Sua5-YciO-YrdC-YwlC family protein |
| *PG1691* | conserved domain protein |
| *PG1692* | ABC transporter, ATP-binding protein |
| *PG1693* | HesA-MoeB-ThiF family protein |
| *PG1694* | conserved hypothetical protein |
| *PG1695* | hypothetical protein |
| *PG1701* | glutamine amidotransferase, class II-dipeptidase |
| *PG1702* | DNA gyrase, B subunit |
| *PG1703* | MazG family protein [only in *Haemophilus influenzae*] |
| *PG1704* | thiol:disulfide interchange protein dsbD, putative |
| *PG1705* | ribosomal large subunit pseudouridine synthase family protein |
| *PG1706* | hypothetical protein |
| *PG1707* | hypothetical protein [septum initiator family] |
| *PG1712* | alpha-1,2-mannosidase family protein |
| *PG1713* | lipoprotein, putative |
| *PG1714* | pyridoxamine-phosphate oxidase |
| *PG1715* | hypothetical protein [Cna protein B-type domain protein] |
| *PG1718* | hypothetical protein |
| *PG1719* | ABC transporter, ATP-binding protein, MsbA family |
| *PG1720* | conserved domain protein |
| *PG1721* | ribonuclease R |
| *PG1722* | hypothetical protein |
| *PG1723* | ribosomal protein S20 |
| *PG1724* | O-sialoglycoprotein endopeptidase |
| *PG1725* | competence-damage-inducible protein CinA domain protein |
| *PG1726* | PDZ domain protein |
| *PG1727* | yitL protein |
| *PG1728* | cytidine-deoxycytidylate deaminase family protein |
| *PG1729* | thiol peroxidase [only in *Vibrio cholerae*] |
| *PG1730* | O-methyltransferase family protein |
| *PG1731* | 3-dehydroquinate dehydratase, type II |
| *PG1732* | integrase-recombinase XerD |
| *PG1733* | hypothetical protein |
| *PG1734* | transporter, putative |
| *PG1739* | conserved domain protein [only in *Mycobacterium tuberculosis*] |
| *PG1741* | aspartate ammonia-lyase |
| *PG1743* | 2-dehydro-3-deoxyphosphooctonate aldolase |
| *PG1745* | phosphoribulokinase family protein [only in *Mycoplasma genitalium*] |
| *PG1747* | ribose 5-phffosphate isomerase B, putative |
| *PG1748* | transketolase |
| *PG1750* | alpha-1,3-4-fucosidase, putative |
| *PG1751* | aminotransferase, class V |
| *PG1752* | hypothetical protein |
| *PG1753* | selenide, water dikinase [only in *E. coli*] |
| *PG1754* | conserved domain protein |
| *PG1755* | fructose-bisphosphate aldolase, class I |
| *PG1758* | ribosomal protein S15 |
| *PG1760* | ABC transporter, ATP-binding protein |
| *PG1761* | acetyltransferase, GNAT family [only in *S. aureus*] |
| *PG1762* | protein-export membrane protein SecD-protein-export membrane protein SecF |
| *PG1763* | ribonuclease III |
| *PG1764* | 3-oxoacyl-(acyl-carrier-protein) synthase II |
| *PG1765* | acyl carrier protein |
| *PG1766* | phosphoribosylglycinamide formyltransferase |
| *PG1767* | lipoprotein, putative [antioxidant, AhpC/TSA family] |
| *PG1768* | magnesium chelatase, subunit D-I family |
| *PG1769* | hypothetical protein |
| *PG1770* | hypothetical protein |
| *PG1771* | phenylalanyl-tRNA synthetase, alpha subunit |
| *PG1772* | endonuclease III [only in *Haemophilus influenzae*] |
| *PG1773* | PAP2 superfamily protein |
| *PG1774* | transcription-repair coupling factor |
| *PG1775* | grpE protein |
| *PG1776* | dnaJ protein |
| *PG1778* | UDP-2,3-diacylglucosamine hydrolase |
| *PG1779* | conserved hypothetical protein [marco domain protein] |
| *PG1780* | 8-amino-7-oxononanoate synthase |
| *PG1781* | uridine kinase |
| *PG1782* | hypothetical protein [glycoside transferase, family 2] |
| *PG1783* | glycosyl transferase, group 2 family protein |
| *PG1784* | conserved hypothetical protein [polysaccharide deacetylase] |
| *PG1787* | hypothetical protein [traJ] |
| *PG1788* | cysteine peptidase, putative |
| *PG1789* | peptidyl-dipeptidase Dcp |
| *PG1790* | hypothetical protein |
| *PG1791* | hypothetical protein |
| *PG1792* | sodium-hydrogen antiporter |
| *PG1793* | 1,4-alpha-glucan branching enzyme |
| *PG1794* | DNA polymerase type I |
| *PG1795* | hypothetical protein |
| *PG1797* | DNA-binding response regulator-sensor histidine kinase |
| *PG1798* | immunoreactive 46 kDa antigen PG99 |
| *PG1799* | hypothetical protein |
| *PG1801* | v-type ATPase, subunit E, putative |
| *PG1802* | hypothetical protein |
| *PG1803* | v-type ATPase, subunit A |
| *PG1804* | v-type ATPase, subunit B |
| *PG1805* | v-type ATPase, subunit D |
| *PG1806* | v-type ATPase, subunit I |
| *PG1807* | v-type ATPase, subunit K |
| *PG1808* | guanosine-3,5-bis(diphosphate) 3-pyrophosphohydrolase [stringent factor] |
| *PG1809* | 2-oxoglutarate oxidoreductase, gamma subunit [only in *Helicobacter pylori*] |
| *PG1810* | 2-oxoglutarate oxidoreductase, beta subunit [only in *Helicobacter pylori*] |
| *PG1811* | hypothetical protein |
| *PG1812* | 2-oxoglutarate oxidoreductase, alpha subunit [only in *Helicobacter pylori*] |
| *PG1813* | ferredoxin, 4Fe-4S |
| *PG1815* | 3-deoxy-D-manno-octulosonate cytidylyltransferase |
| *PG1816* | NAD(P)H dehydrogenase, quinone family, putative |
| *PG1817* | conserved hypothetical protein [cytochrome C assembly] |
| *PG1818* | hypothetical protein [respiratory nitrite reductase cytochrome assembly] |
| *PG1819* | hypothetical protein [respiratory nitrite reductase cytochrome assembly] |
| *PG1820* | cytochrome c nitrite reductase, catalytic subunit NrfA [only in *Haemophilus influenzae*] |
| *PG1821* | cytochrome c nitrite reductase, small subunit NrfH |
| *PG1823* | hypothetical protein [outer membrane protein] |
| *PG1824* | Glycogen synthase |
| *PG1825* | hypothetical protein |
| *PG1826* | conserved domain protein |
| *PG1827* | RNA polymerase sigma-70 factor, ECF subfamily |
| *PG1828* | lipoprotein, putative |
| *PG1829* | long-chain-fatty-acid--CoA ligase, putative |
| *PG1831* | ATP-dependent DNA helicase RecQ |
| *PG1834* | glycogen synthase-related protein |
| *PG1835* | lipoprotein, putative |
| *PG1836* | nucleoside permease NupG |
| *PG1837* | hemagglutinin protein HagA |
| *PG1840* | conserved domain protein |
| *PG1841* | conserved hypothetical protein |
| *PG1842* | acetyltransferase, GNAT family |
| *PG1844* | hemagglutinin protein HagD |
| *PG1847* | endoribonuclease L-PSP, putative |
| *PG1848* | RNA methyltransferase, TrmH family |
| *PG1849* | DNA repair protein RecN |
| *PG1850* | hypothetical protein |
| *PG1851* | phosphopantothenoylcysteine decarboxylase-phosphopantothenate--cysteine ligase |
| *PG1852* | DNA polymerase III epsilon chain |
| *PG1853* | DNA polymerase III, beta subunit |
| *PG1854* | 5-formyltetrahydrofolate cyclo-ligase family protein [only in *Haemophilus influenzae*] |
| *PG1855* | carboxyl-terminal protease [only in *Salmonella*] |
| *PG1856* | cytidine-deoxycytidylate deaminase family protein |
| *PG1857* | conserved hypothetical protein |
| *PG1858* | flavodoxin |
| *PG1859* | glycerate kinase family protein [only in *Haemophilus influenzae*] |
| *PG1860* | conserved hypothetical protein [ATPase, AAA family] |
| *PG1861* | hypothetical protein |
| *PG1862* | hypothetical protein |
| *PG1863* | hypothetical protein |
| *PG1868* | membrane protein, putative |
| *PG1871* | hypothetical protein |
| *PG1874* | conserved hypothetical protein |
| *PG1875* | hemolysin |
| *PG1876* | conserved hypothetical protein |
| *PG1878* | cysteinyl-tRNA synthetase |
| *PG1879* | conserved hypothetical protein [patatin] |
| *PG1880* | glycosyl transferase, group 2 family protein |
| *PG1881* | hypothetical protein [lipoprotein] |
| *PG1884* | alpha-L-fucosidase precursor, putative |
| *PG1885* | polyphosphate kinase |
| *PG1886* | GTP-binding protein HflX |
| *PG1887* | rhodanese-like domain protein |
| *PG1895* | hypothetical protein |
| *PG1896* | S-adenosylmethionine synthase |
| *PG1897* | thiamine pyrophosphokinase |
| *PG1898* | transporter, putative |
| *PG1899* | TonB-dependent receptor, putative |
| *PG1900* | conserved hypothetical protein [ribosome-associated GTPase] |
| *PG1901* | ribosome recycling factor |
| *PG1902* | uridylate kinase |
| *PG1903* | conserved hypothetical protein |
| *PG1904* | hypothetical protein |
| *PG1910* | ribosomal protein L17 |
| *PG1911* | DNA-directed RNA polymerase, alpha subunit |
| *PG1912* | ribosomal protein S4 |
| *PG1913* | ribosomal protein S11 |
| *PG1914* | ribosomal protein S13 |
| *PG1915* | ribosomal protein L36 |
| *PG1916* | translation initiation factor IF-1 |
| *PG1917* | methionine aminopeptidase, type I |
| *PG1918* | preprotein translocase, SecY subunit |
| *PG1919* | ribosomal protein L15 |
| *PG1920* | ribosomal protein L30 |
| *PG1921* | ribosomal protein S5 |
| *PG1922* | ribosomal protein L18 |
| *PG1923* | ribosomal protein L6 |
| *PG1924* | ribosomal protein S8 |
| *PG1925* | ribosomal protein S14 |
| *PG1926* | ribosomal protein L5 |
| *PG1927* | ribosomal protein L24 |
| *PG1928* | ribosomal protein L14 |
| *PG1929* | ribosomal protein S17 |
| *PG1930* | ribosomal protein L29 |
| *PG1931* | ribosomal protein L16 |
| *PG1932* | ribosomal protein S3 |
| *PG1933* | ribosomal protein L22 |
| *PG1934* | ribosomal protein S19 |
| *PG1935* | ribosomal protein L2 |
| *PG1936* | ribosomal protein L23 |
| *PG1937* | ribosomal protein L4 |
| *PG1938* | ribosomal protein L3 |
| *PG1939* | ribosomal protein S10 |
| *PG1940* | translation elongation factor G |
| *PG1941* | ribosomal protein S7 |
| *PG1942* | ribosomal protein S12 |
| *PG1943* | hypothetical protein |
| *PG1944* | 3-phosphoshikimate 1-carboxyvinyltransferase [only in *Caulobacter crescentus*] |
| *PG1945* | hypothetical protein [phospholipase A2] |
| *PG1946* | ABC 3 transporter family protein |
| *PG1947* | TPR domain protein |
| *PG1948* | lipoprotein, putative |
| *PG1949* | malate dehydrogenase |
| *PG1950* | membrane protein, putative |
| *PG1951* | glutaminyl-tRNA synthetase |
| *PG1952* | DedA family protein |
| *PG1953* | YitT family protein |
| *PG1954* | NAD dependent epimerase-reductase-related protein |
| *PG1956* | 4-hydroxybutyrate CoA-transferase |
| *PG1959* | ribosomal protein L33 |
| *PG1960* | ribosomal protein L28 |
| *PG1961* | hypothetical protein |
| *PG1963* | Sua5-YciO-YrdC-YwlC family protein |
| *PG1964* | bacterial sugar transferase [only in *Streptococcus pneumoniae*] |
| *PG1965* | voltage gated chloride channel, authentic frameshift |
| *PG1966* | conserved hypothetical protein [transport protein] |
| *PG1967* | TPR domain protein |
| *PG1992* | glucose-inhibited division protein A |
| *PG1993* | excinuclease ABC, C subunit |
| *PG1994* | D-tyrosyl-tRNA(Tyr) deacylase [only in *S. aureus*] |
| *PG1995* | conserved hypothetical protein [mazG nucleotide pyrophosphohydrolase] |
| *PG1996* | deoxyribose-phosphate aldolase |
| *PG1997* | hypothetical protein |
| *PG1998* | polyprenyl synthetase |
| *PG1999* | conserved hypothetical protein |
| *PG2000* | signal peptidase-related protein |
| *PG2001* | signal peptidase I |
| *PG2002* | dihydrodipicolinate reductase |
| *PG2003* | deoxyguanosinetriphosphate triphosphohydrolase |
| *PG2004* | conserved hypothetical protein [only in *Haemophilus influenzae*] |
| *PG2006* | hypothetical protein [membrane protein] |
| *PG2008* | TonB-dependent receptor, putative [only in *Helicobacter pylori*] |
| *PG2009* | DNA repair protein RecO, putative |
| *PG2010* | phosphomannomutase, putative |
| *PG2013* | CRISPR-associated protein Cas2 |
| *PG2014* | CRISPR-associated protein Cas1 [only in *Mycobacterium tuberculosis*] |
| *PG2015* | CRISPR-associated protein Cas4 |
| *PG2020* | CRISPR-associated protein, TM1814 family |
| *PG2021* | conserved hypothetical protein [arylsulfatase] [only in *Haemophilus influenzae*] |
| *PG2022* | hypothetical protein |
| *PG2023* | methionyl-tRNA formyltransferase |
| *PG2026* | phosphoglycerate mutase family protein |
| *PG2027* | hypothetical protein |
| *PG2028* | ebsC protein |
| *PG2029* | hypothetical protein [peptidase M10A and M12B matrixin and adamalysin] |
| *PG2030* | hypothetical protein |
| *PG2031* | hypothetical protein |
| *PG2032* | primosomal protein N |
| *PG2033* | glutamate synthase, small subunit |
| *PG2034* | oxidoreductase, FAD-binding, putative |
| *PG2035* | tRNA (guanine-N1)-methyltransferase |
| *PG2036* | ion transporter [only in *Mycoplasma pulmonis*] |
| *PG2037* | hypothetical protein |
| *PG2038* | N-acetylmuramoyl-L-alanine amidase, putative [only in *Haemophilus influenzae*] |
| *PG2040* | DNA-binding protein, histone-like family |
| *PG2041* | hypothetical protein [outer membrane protein] |
| *PG2043* | conserved hypothetical protein [dinuclear metal center protein] |
| *PG2044* | conserved hypothetical protein |
| *PG2046* | tRNA(Ile)-lysidine synthetase |
| *PG2047* | helicase, putative |
| *PG2048* | hypothetical protein |
| *PG2049* | hypothetical protein |
| *PG2050* | hypothetical protein |
| *PG2052* | dihydrodipicolinate synthase |
| *PG2053* | dethiobiotin synthase |
| *PG2054* | lipoprotein PG3 [only in *Vibrio cholerae*] |
| *PG2055* | dihydroorotate dehydrogenase family protein |
| *PG2056* | transposase, ISPg2-related, truncation |
| *PG2060* | thymidylate synthase |
| *PG2061* | dihydrofolate reductase |
| *PG2062* | histidyl-tRNA synthetase |
| *PG2064* | hypothetical protein |
| *PG2065* | conserved hypothetical protein [ribosomal RNA large subunit methyltransferase N] |
| *PG2066* | lipoprotein, putative |
| *PG2067* | pyridoxal phosphate biosynthetic protein PdxA |
| *PG2068* | glycerol-3-phosphate cytidylyltransferase |
| *PG2069* | oxidoreductase, short chain dehydrogenase-reductase family |
| *PG2070* | hypothetical protein [CDP-alcohol phosphatidyltransferase] |
| *PG2071* | conserved domain protein |
| *PG2072* | UvrD-REP helicase domain protein |
| *PG2078* | conserved hypothetical protein [Archaeal ATPase] |
| *PG2079* | hypothetical protein |
| *PG2080* | adenosylmethionine--8-amino-7-oxononanoate aminotransferase |
| *PG2081* | biotin synthetase |
| *PG2083* | hypothetical protein |
| *PG2085* | tryptophanyl-tRNA synthetase |
| *PG2086* | hypothetical protein |
| *PG2088* | peptide methionine sulfoxide reductase |
| *PG2089* | hypothetical protein [DNA helicase] |
| *PG2090* | cation efflux family protein [only in *Streptococcus pneumoniae*] |
| *PG2092* | hypothetical protein [apaG] |
| *PG2094* | conserved domain protein |
| *PG2095* | lipoprotein, putative [outer membrane protein] |
| *PG2096* | conserved domain protein |
| *PG2097* | ribose-phosphate pyrophosphokinase |
| *PG2099* | ATP-dependent RNA helicase, DEAD-DEAH box family |
| *PG2101* | hypothetical protein |
| *PG2102* | immunoreactive 61 kDa antigen PG91 |
| *PG2103* | hypothetical protein |
| *PG2104* | hypothetical protein |
| *PG2105* | lipoprotein, putative |
| *PG2106* | hypothetical protein [outer membrane protein] |
| *PG2107* | thiH protein |
| *PG2108* | thiG protein |
| *PG2110* | thiamine biosynthesis protein ThiC |
| *PG2111* | thiamine biosynthesis protein ThiS |
| *PG2116* | hypothetical protein |
| *PG2117* | ribosomal protein S16 |
| *PG2119* | oxidoreductase, Gfo-Idh-MocA family |
| *PG2120* | metallo-beta-lactamase superfamily protein [only in *S. aureus*] |
| *PG2121* | L-asparaginase [only in *E. coli*] |
| *PG2122* | ISPg2, transposase, truncation |
| *PG2123* | hypothetical protein |
| *PG2124* | glyceraldehyde 3-phosphate dehydrogenase, type I |
| *PG2125* | transcriptional regulator, AraC family |
| *PG2126* | conserved hypothetical protein TIGR00044 [pyridoxal phosphate enzyme] |
| *PG2127* | hypothetical protein |
| *PG2130* | hypothetical protein |
| *PG2131* | 60 kDa protein |
| *PG2133* | lipoprotein, putative |
| *PG2139* | hypothetical protein [riboflavin synthase subunit alpha] |
| *PG2140* | ribosomal protein L32 |
| *PG2141* | 3-oxoacyl-(acyl-carrier-protein) synthase III |
| *PG2142* | GTP-binding protein Era |
| *PG2143* | GTP-binding protein, Era-ThdF family |
| *PG2144* | hypothetical protein |
| *PG2145* | polysaccharide deacetylase |
| *PG2146* | conserved hypothetical protein |
| *PG2147* | xanthine phosphoribosyltransferase |
| *PG2148* | xanthine-uracil permease family protein [only in *S. aureus*] |
| *PG2149* | hypothetical protein [outer membrane protein] |
| *PG2150* | LysM domain protein |
| *PG2153* | transposase, truncation |
| *PG2154* | hypothetical protein |
| *PG2155* | lipoprotein, putative |
| *PG2156* | conserved hypothetical protein [only in *Haemophilus influenzae*] |
| *PG2157* | glutamine cyclotransferase-related protein |
| *PG2158* | SufE Fe/S-cluster-related protein |
| *PG2159* | protoporphyrinogen oxidase |
| *PG2161* | transcriptional regulator, AraC family |
| *PG2162* | lipid A disaccharide synthase |
| *PG2163* | stationary-phase survival protein SurE |
| *PG2164* | peptidyl-prolyl cis-trans isomerase, FKBP-type |
| *PG2165* | glycyl-tRNA synthetase |
| *PG2167* | immunoreactive 53 kDa antigen PG123 |
| *PG2168* | hypothetical protein [fimX] |
| *PG2170* | sugar transporter |
| *PG2171* | D-isomer specific 2-hydroxyacid dehydrogenase family protein |
| *PG2173* | outer membrane lipoprotein Omp28 |
| *PG2174* | hypothetical protein |
| *PG2175* | conserved hypothetical protein [antioxidant, AhpC/TSA family][only in *Bacillus subtilis*] |
| *PG2177* | NADH:ubiquinone oxidoreductase, Na translocating, F subunit [only in *Francisella novicida*] |
| *PG2178* | NADH:ubiquinone oxidoreductase, Na translocating, E subunit |
| *PG2179* | NADH:ubiquinone oxidoreductase, Na(+)-translocating, D subunit |
| *PG2180* | NADH:ubiquinone oxidoreductase, Na translocating, C subunit |
| *PG2181* | NADH:ubiquinone oxidoreductase, Na translocating, B subunit [only in *Haemophilus* *influenzae*] |
| *PG2186* | transcriptional regulator, putative |
| *PG2187* | 1,4-dihydroxy-2-naphthoate octaprenyltransferase |
| *PG2188* | diaminopimelate decarboxylase |
| *PG2189* | aspartate kinase |
| *PG2190* | cell-division ATP-binding protein |
| *PG2192* | peptidase, M23-M37 family |
| *PG2195* | ISPg1, transposase, truncation |
| *PG2197* | conserved hypothetical protein [peptidase M48 family] [only in *Salmonella*] |
| *PG2198* | hemagglutinin protein, truncation |
| *PG2199* | ABC transporter, ATP-binding protein, putative |
| *PG2200* | TPR domain protein |
| *PG2201* | peptide deformylase |
| *PG2202* | conserved hypothetical protein TIGR00250 |
| *PG2204* | hypothetical protein |
| *PG2205* | 2-dehydropantoate 2-reductase |
| *PG2206* | ABC transporter, ATP-binding protein |
| *PG2207* | conserved domain protein |
| *PG2210* | excinuclease ABC, A subunit |
| *PG2212* | hypothetical protein |
| *PG2213* | nitrite reductase-related protein |
| *PG2214* | hypothetical protein |
| *PG2215* | mannose-1-phosphate guanylyltransferase |
| *PG2216* | hypothetical protein |
| *PG2217* | deoxyxylulose-5-phosphate synthase |
| *PG2218* | potassium uptake protein TrkA |
| *PG2219* | potassium uptake protein TrkH |
| *PG2220* | hypothetical protein [traJ] |
| *PG2221* | MiaB-like tRNA modifying enzyme |
| *PG2223* | glycosyl transferase, group 2 family protein |
| *PG2224* | membrane protein, putative |
| *PG2225* | conserved hypothetical protein |
| *PG2226* | hypothetical protein |
| *PG2227* | hypothetical protein |
